# Gemcitabine-induced Gli-dependent activation of hedgehog pathway resists to the treatment of urothelial carcinoma cells

**DOI:** 10.1371/journal.pone.0254011

**Published:** 2021-07-08

**Authors:** Yu-Hao Chang, Hoi-Lam Tam, Meng-Chien Lu, Huei-Sheng Huang

**Affiliations:** Department of Medical Laboratory Science and Biotechnology, College of Medicine, National Cheng Kung University, Tainan, Taiwan; University of Alabama at Birmingham, UNITED STATES

## Abstract

Patients with urothelial carcinoma (UC) experience gemcitabine resistance is a critical issue. The role of hedgehog pathway in the problem was explored. The expressions of phospho-AKT^ser473^, phospho-GSK3β^ser9^ and Gli2 were up-regulated in gemcitabine-resistant NTUB1 (NGR) cells. Without hedgehog ligands, Gli proteins can be phosphorylated by GSK3β kinase to inhibit their downstream regulations. Furthermore, the GSK3β kinase can be phosphorylated by AKT at its Ser9 residue to become an inactive kinase. Therefore, overexpression of *AKT1*, *Flag-GSK*^*S9D*^ (constitutively inactive form) or active *Gli2 (GLI2ΔN)* in NTUB1 cells could activate Gli2 pathway to enhance migration/invasion ability and increase gemcitabine resistance, respectively. Conversely, overexpression of *Flag-GSK*^*S9A*^ (constitutively active form) or knockdown of Gli2 could suppress Gli2 pathway, and then reduce gemcitabine resistance in NGR cells. Therefore, we suggest gemcitabine-activated AKT/GSK3β pathway can elicit Gli2 activity, which leads to enhanced migration/invasion ability and resistance to gemcitabine therapy in UC patients. The non-canonical hedgehog pathway should be evaluated in the therapy to benefit UC patients.

## Introduction

Urothelial carcinoma (UC) of the bladder is estimated the 4th most commonly diagnosed cancer and the 8th most common cancer-related death in males in the United States as reported in 2020 [1]. UC can be classified into 2 subtypes, non-muscle invasive UC (NMIUC) and muscle invasive UC (MIUC). About 70–80% of patients with UC have NMIUC. They have a favorable outcome but a high recurrence rate. Conversely, patients with MIUC have higher rates of metastasis and mortality, and need frequent diagnoses and treatments throughout their remaining life [2]. Generally, the average medical cost per UC patient was estimated to be the most expensive among all cancers [3, 4]. Many risk factors associated with UC were reported, including tobacco smoking, occupational exposure to aromatic amines, consumption of arsenic-laced water, and herbal medicines containing aristolochic acid [5, 6].

The standard treatment for UC is transurethral resection followed by either intravesical chemotherapy or bacillus Calmette-Guerin (BCG) immunotherapy [6]. To reduce the recurrence and progression of UC, systemic chemotherapy is considered. The agents used in the remedy include gemcitabine plus cisplatin (GC), or methotrexate/vinblastine/doxorubicin/cisplatin (MVAC). GC regimens express similar therapeutic effects but less significant toxicities to MVAC regimens for locally advanced and metastatic UC; therefore, these regimens are most commonly used [7]. However, drug-resistance and rapid relapse/recurrence are the major reasons for treatment failure. This reveals the need to understand the GC-resistant mechanisms in order to develop a novel strategy for effective treatment [8].

Gemcitabine (2’, 2’-difluorodeoxycytidine, dFdC) is an anti-metabolic agent and cytidine analog, which has been widely used in antitumor treatment in various cancers, including breast cancer, lung cancer, metastatic pancreatic cancer, and bladder cancer [7]. It can be phosphorylated by deoxycytidine kinase (dCK) after its cellular uptake by human equilibrative nucleoside transporter 1 (hENT1) and the human concentrative nucleoside transporter 3 (hCNT3). Then the gemcitabine is converted into active metabolites to inhibit ribonucleotide reductase (RR), or to be incorporated into DNA to inhibit DNA polymerase, thereby leading to inhibiting DNA synthesis and promoting apoptosis [9]. In addition, gemcitabine also can be deaminated by cytidine deaminase (CDA) to inactivate function. Therefore, it has been reported that silence of hENTs or dCK, or overexpression of CDA or RR can cause gemcitabine resistance [10].

Activation of hedgehog (HH) pathway modulates tumorigenesis in various cancers, including UC [11–13]. Recently, many studies have demonstrated that the HH pathway plays a critical role in the maintenance and progression of cancers, and can become a promising therapeutic target for the development of anticancer agents [14]. In addition, the aberrant HH pathway has also been linked to epithelial-mesenchymal transition (EMT) type cells and cancer stem cells (CSCs) maintenance, which have been suggested to play important roles in metastasis and drug-resistance of cancers. HH pathway activation is initiated by the binding of HH ligands, including desert hedgehog (DHH), Indian hedgehog (IHH), and sonic hedgehog (SHH), to receptor Patched (Ptch) protein. In the absence of the HH ligands, Ptch catalytically inhibits the activity of the smoothened (SMO) by preventing its localization to the cell surface. Under these circumstances, PKA, GSK3β or CKIα can phosphorylate Gli2/3 to become a repressor form Gli2/3R, which then translocates to the nucleus to repress target gene expression.

In the canonical HH pathway, binding of the HH ligands can release the inhibitory effects on SMO and increase its translocation to the primary cilia, which then dissociates suppressor of Fused (SUFU) and Gli within the cilia. The activated Gli proteins then translocate to the nucleus to induce target gene expression [14, 15]. Moreover, the HH pathway can also be activated via non-canonical pathways. Increasing evidence suggests that non-canonical HH signaling also plays important roles in tumorigenesis, development, and drug-resistance, including Gli-independent activation and HH ligands or Ptch/SMO-independent activation [16, 17].

In this study, we demonstrated that gemcitabine-induced AKT activation could inactivate GSK3β kinase to elicit non-canonical Gli2-dependent HH pathway, which promoted migration/invasion abilities and resistance to gemcitabine treatment in UC cells.

## Materials and methods

### Reagents and antibodies

RPMI 1640 medium, Opti-MEM medium, and fetal bovine serum (FBS) were obtained from Invitrogen (Carlsbad, CA). Hyfect^TM^ DNA transfection reagent was from Leadgene (Tainan, Taiwan). Luciferase assay system and GoTaq^®^ Green Master Mix (2X) were from Promega (Madison, WI). DNA Polymerase Premix (2X) was from Yeastern Biotech (Taipei, Taiwan). Antibodies against phospho-AKT^Ser473^, AKT, phospho-GSK3β^Ser9^, GSK3α/β, and Gli2 were from Santa Cruz Biotechnology (Santa Cruz, CA). Anti-*β*-actin antibody was from Sigma-Aldrich (St. Louis, MO). The *pCS2MT-Gli2ΔN* plasmid (active form of Gli2) was a gift from Erich Roessler (the contributor of the plasmid to Addgene; Cambridge, MA). The *pLKO*.*1-Gli2-shRNA* (targets: 5´-CCGCTTCAGATGACAGATGTT-3´; 5´-GTTCCTGAACATGATGACCTA-3´; 5´-GCTCTACTACTACGGCCAGAT-3´; 5´-CCAACGAGAAACCCTACATCT-3´), and luciferase control (*pLKO*.*1-shLuc*) were obtained from National RNAi Core Facility located at the Institute of Molecular Biology/Genomic Research Center, Academia Sinica (Taipei, Taiwan). The *pcTGIF* plasmids were cloned from human TGIF1 [18]. As described previously [19], the *Flag-GSK*^*S9A*^ (constitutively active GSK3β) and *Flag-GSK*^*S9D*^ (constitutively inactive GSK3β) plasmids were constructed by using site-directed mutagenesis according to the manufacturer’s instruction of GeneTailor. The *pcHA-AKT1* plasmids were cloned from the human *AKT1* genes to ligate into the *pcDNA-HA* (+) expression vector containing the *CMV* promoter and validated by sequencing [20].

### Cell culture and transfection

Human urothelial carcinoma cell lines (NTUB1 and NGR) were kindly from Dr. Yu. The NTUB1 cells were derived from the surgical specimen of a Taiwanese patient with poorly differentiated transitional cell carcinoma [21]. Then the NTUB1 cells were chronically exposed to progressively increasing concentrations of gemcitabine to cause gemcitabine-resistance. A subline that could survive in 1.5 μM gemcitabine was established and designated as NGR [22]. We also cultured T24 cell with 10 nM gemcitabine at the beginning, and gradually increased the concentration of gemcitabine in medium once a week for 6 months. Finally, we generated a gemcitabine resistant T24 cells, which could survive in 1000 nM gemcitabine and designated as T24_GR. These cells were cultured in RPMI 1640 medium supplemented with 10% FBS, 100 units/ml penicillin, and 100 μg/ml streptomycin (Gibco BRL, Grand Island, NY) in a highly humidified atmosphere of 5% CO_2_ at 37 °C.

Cellular transfection method was based on the manufacturer instruction of HyFect^TM^ DNA transfection reagent with a slight modification as described follows. Cells (3 x 10^5^) were seeded into 6-cm culture plate for 24 h. Plasmids and HyFect^TM^ DNA transfection reagent were mixed in 0.6 ml of Opti-MEM medium, and then incubated at RT for 20 min. The mixture was added into cells to incubate at 37°C for another 24 h. Then the cells were lysed for the determination of luciferase activities and protein expression, respectively.

### Cell viability assay

Cells (1 x 10^5^) were seeded in each well of a 96-well plate (Falcon; USA) with RPMI 1640 containing 10% FBS. After 24 h, culture medium was exchanged to RPMI 1640 with 10% FBS and gemcitabine (0−0.4 μM), and the cells were incubated for another 72 h. Thereafter, the number of cells was quantified by using a counting chamber. The experiment was replicated 3 times, and the cell viability percentage was normalized with the control.

### Quantitative real-time PCR analysis

Total RNA was isolated from NTUB1 and NGR cells by using RNAzol® RT RNA isolation reagent (Molecular Research Center) according to the manufacture instructions. Reverse transcription of total RNA (5 μg) was performed by using MMLV reverse transcription kit (Promega). Gene expression levels were quantitatively measured by using StepOnePlus™ Real-Time PCR System (Applied Biosystem), and calculated by the 2^-ΔΔCt^ method. The relative expression level of each candidate gene was normalized with *β*-actin. The specific primers (dCK sense: 5′-GCTGCAGGGAAGTCAACATTT-3′ and antisense: 5′-TTCAGGAACCACTTCCCAATC-3′; hENT1 sense: 5′-GCAAAGGAGAGGAGCCAAGAG-3′ and antisense: 5′-GGGCTGAGAGTTGG AGACTG-3′; *β*-actin sense: 5′-TCCCTGGAGAAGAGCTACGA-3′ and antisense: 5′-ACTCCATG CCCAGGAAGG-3′)

### Western blot

Thirty μg of protein were subjected to 10% SDS-PAGE to run polyacrylamide gel electrophoresis (PAGE) to separate individual proteins, and then transferred onto PVDF membrane (IPVH00010; Millipore) on a semi-dry transfer apparatus (Hoefer). Then the PVDF membrane was incubated with primary antibodies dCK (Abcam; ab96599, Lot: GR3303586-1), hENT1 (Sigma-Aldrich; B5500117, Lot: GR3217413-6), pAKT^Ser473^ (Cell Signaling Technology; #4060, Lot: 25), AKT (Cell Signaling Technology; #9272, Lot: 27), pGSK3β^Ser9^ (Cell Signaling Technology; #9336, Lot: 13), GSK3α/β (Santa Cruz; SC-7291), and Gli2 (Invitrogen; #PA5-79314, Lot: VG3044693) at 4°C overnight for immunoblotting. The *β*-actin (Santa Cruz; SC-47778) and *β*-tubulin (Cell Signaling Technology; #2146) was served as a loading control. Anti-rabbit IgG (Jackson ImmunoResearch; #118578) or anti-mouse IgG (Jackson ImmunoResearch; #120646) antibody conjugated with horseradish peroxidase (HRP) was used as a secondary antibody. The protein expression was developed by using an enhanced chemiluminescence kit (Amersham), and then detected by Fujifilm LAS-3000 imager. Quantitation of the results was carried out by an image analysis system installed with a software UN-SCAN-IT gel 6.1.

### DNA construct and reporter assay

For the *Gli* responsive element (*Gli-luc*) construction, two oligonucleotides containing 8-*Gli* binding sites (5′-GGTACCGACCACCCAGACCACCCAGACCACCCAGACCACCCAGACCACC CAGACCACCCAGACCACCCAGACCACCCAAGATCT-3′ and 5′-AGATCTTGGGTGGTCTGG GTGGTCTGGGTGGTCTGGGTGGTCTGGGTGGTCTGGGTGGTCTGGGTGGTCTGGGTGGTCGGTACC-3′) were annealed. The *Gli* responsive element was then cloned by using PCR with two specific primers (sense: 5′-GCCATGGCTGGTGGGTCTGGT-3′ and antisense: 5′-GGGAGATCTTGGGTGGTCTGGG-3′) and the above annealed oligonucleotides as a template. The PCR products were cloned into T&A cloning vector, confirmed by DNA sequencing, and then sub-cloned into the Kpn I/Bgl II-digested pGL3-basic (Promega). The *Gli-luc* reporter was used as an indicator of HH pathway activation.

Cells were sub-cultured in a 12-well plate at a density of 8 x 10^4^ cells/well with 0.5 ml culture medium for 24 h. After transfection with *Gli-luc* alone or with other plasmids for another 48 h, the cells were lysed by the lysis buffer for the determination of luciferase activities as described previously [23]. The values of luciferase activity were measured by FB12 Luminometer (Zylux Corporation, Huntsville, AL). The luciferase activity was determined and normalized by the amount of total protein. Values are means±SD for three determinations.

### Cellular migration/invasion assay

Cellular migration/invasion assay was performed by using a 6.5 mm Transwell^®^ chamber with 8-μm pore size (Corning, Corning, NY). After transfection with *Gli2ΔN* plasmids for 48 h, cells were harvested and re-suspended in serum-free medium, then the cells (5 x 10^5^) were seeded onto the upper chamber with uncoated polycarbonate membrane for migration assay, or with Matrigel-coated (BD Bioscience, Bedford, MA) membrane for invasion assay, respectively. After 24 h incubation at 37°C, cells on the upper side of membrane were removed by a cotton swab. The migrating cells onto the bottom surface of the membrane were fixed with 100% methanol for 10 min, stained with 10% Giemsa for 30 min, and counted under a microscope in 5 random fields (100 X) per well, and then quantified by a software Image-J. Values are mean ± SD for three determinations.

### Statistical analysis

All experiments were performed for at least 3 times. Statistical analysis was performed by using the unpaired Student’s t-test of Microsoft Excel™ statistics in cellular experiments. The values were presented as mean ± SD. (*p < 0.05, **p < 0.01, ***p < 0.001).

## Results

### High expression of phospho-AKT^Ser473^, phospho-GSK3β^Ser9^ and Gli2 in NGR and T24_GR cells

**Fig 1:**
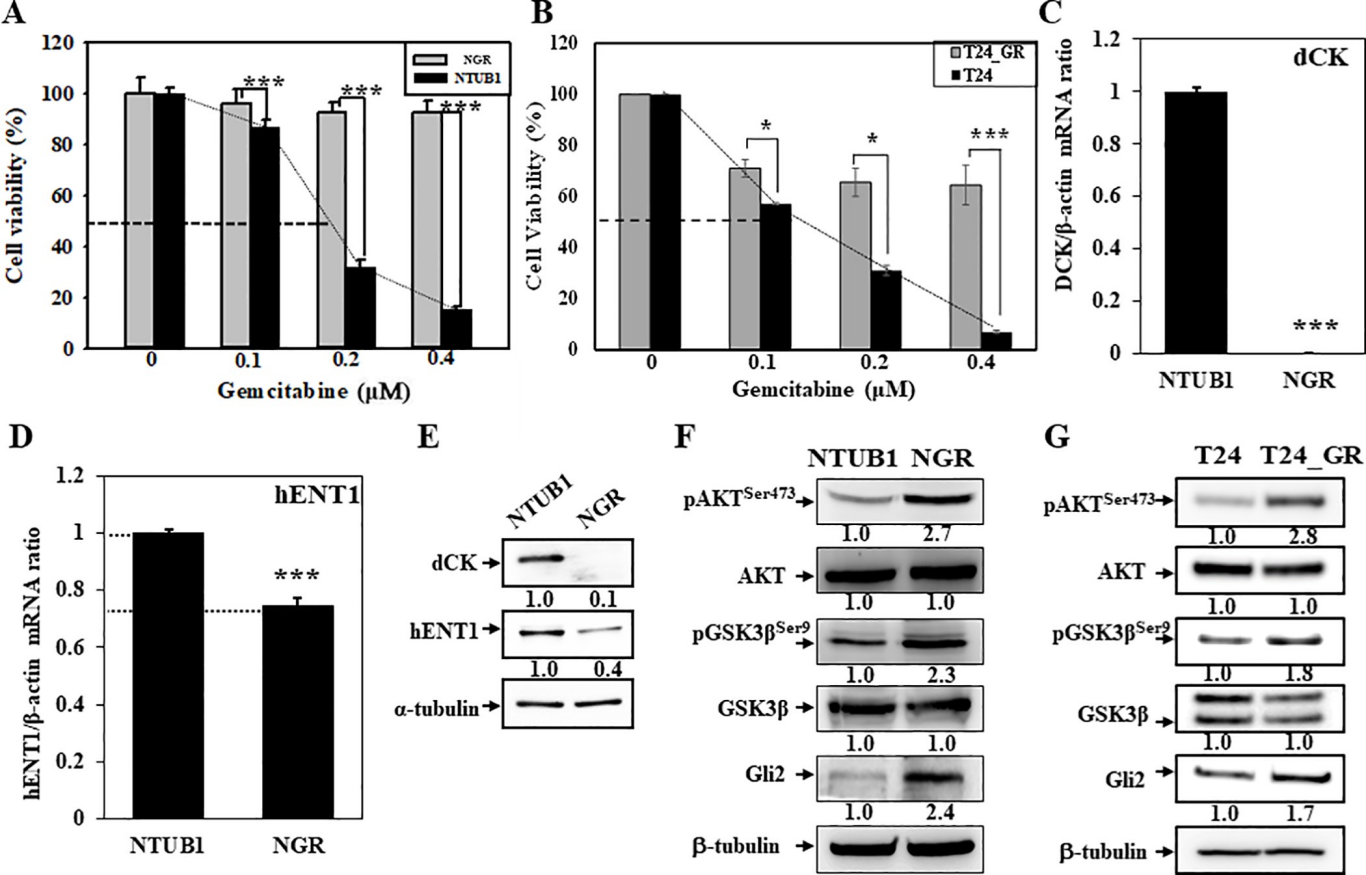
Increased phospho-AKT^Ser473^, phospho-GSK3β^Ser9^ and Gli2 proteins expression in NGR and T24_GR cells. (A) NTUB1/NGR and (B) T24/T24_GR cells were treated with various doses of gemcitabine (0−0.4 μM) for 72 h, and were trypsinized and counted numbers by using a counting chamber to measure cellular viability. (C, D) Total RNA or (E-G) cell lysates were collected from NTUB1/NGR or T24/T24_GR cells to perform (C, D) quantitative real-time PCR or (E-G) Western blot, respectively. Gemcitabine resistance-related genes dCK and hENT1 were measured at (C, D) mRNA or (E) protein levels, respectively. Protein expression of phopho-AKT^ser473^, phospho-GSK3β^ser9^ and Gli2 was also detected in (F) NTUB1/NGR and (G) T24/T24_GR cells, respectively.

To explore the molecular mechanism of gemcitabine-induced resistance in UC cells, we compared the gemcitabine-resistant NGR cells with their parental cells NTUB1. As shown in the cell viability assay, the response to gemcitabine in NTUB1 was more sensitive than that in NGR cells after treatment with various doses of gemcitabine (0–0.4 μM) for 72 h (Fig 1A). The estimated IC_50_ for NTUB1 was about 0.15~0.20 μM, but the NGR cells still grew well in the same concentrations. In addition, we also generated a gemcitabine resistant T24 cell designated as T24_GR. The estimated IC_50_ for T24 was about 0.1~0.15 μM (Fig 1B). The T24_GR cells were also more resistant to gemcitabine treatment than T24 cells. The gemcitabine resistance-related genes dCK and hENT1 were down-regulated in NGR cells at mRNA (Fig 1C and 1D) and protein levels (Fig 1E), respectively, which is consistent with the previous report [10]. As expected, the protein levels of phospho-AKT^Ser473^, phospho-GSK3β^Ser9^, and Gli2 were up-regulated in NGR and T24_GR cells (Fig 1F and 1G), respectively.

### Involvement of AKT/GSK3β pathway in Gli2 activation in UC cells

**Fig 2:**
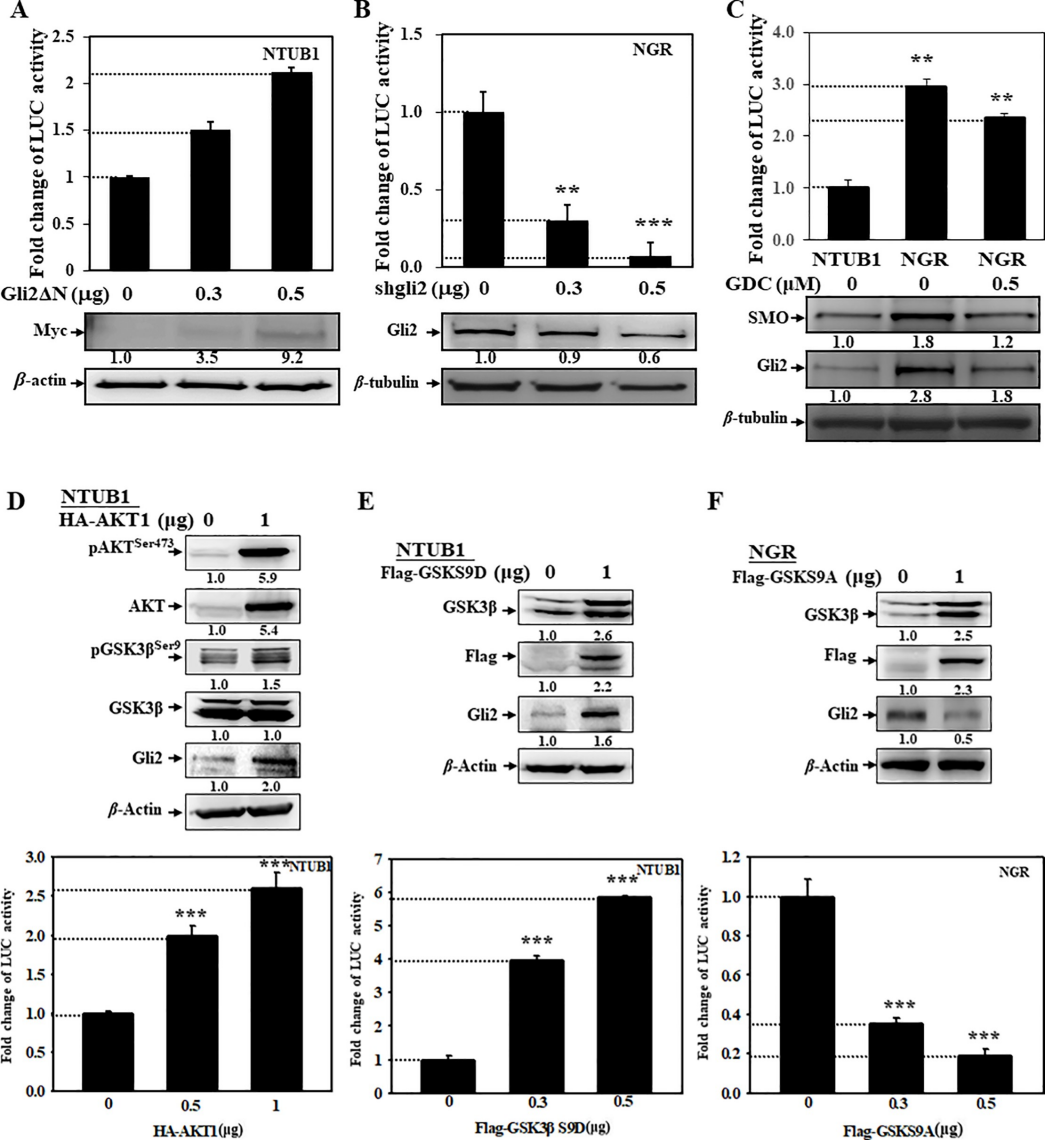
AKT/GSK3β pathway regulated Gli2 activation in UC cells. Cells were co-transfected with reporters and (A) *pCS2MT-Gli2ΔN*, (B) Gli2 specific shRNA plasmids, (D-F) *pcAKT*, *Flag-GSK*^*S9D*^ (constitutively inactive GSK-3β), or *Flag-GSK*^*S9A*^ (constitutively active GSK-3β) plasmids, respectively, and then harvested for the detection of their *Gli2-luc* activity and protein expression. (C) Cells were transfected with *Gli-luc* followed by treatment with SMO specific inhibitor, GDC0449, for 24 h. The *Gli2-luc* activity was measured as described in the “Materials and Methods” section.

To clarify the HH pathways in the gemcitabine resistance, the *Gli-luc* reporter was constructed as described in the “Materials and Methods” section. Overexpression of *Gli2ΔN* (active form of Gli2) could increase *Gli-luc* activity in NTUB1 cells (Fig 2A) and in T24 cells (S1A Fig). Thus, we demonstrated the reporter could be used in the measurement of Gli2 activation. In addition, knockdown of Gli2 by its specific shRNA in NGR cells could decrease *Gli-luc* activity (Fig 2B). To reduce the off-target effects, we cotransfected 4 specific shRNAs with different targets to Gli2 in NGR cells, and used pLKO.1-shLuc vector as a control. The profile of inhibition was similar to that in Fig 2B (S1B Fig). Furthermore, we performed rescue experiments to demonstrate that the results are not due to off-target effects (S1C Fig). Therefore, the NTUB1 and NGR cells were compared with their HH pathway activation. As shown in the Fig 2C, the SMO, Gli2 expression, and *Gli-luc* activity in NGR cells were higher than those in NTUB1 cells. However, after treatment with SMO specific inhibitor, GDC0449, the Gli2 activation was only partially inhibited (Fig 2C). Therefore, we suggest that there might be SMO-independent HH pathway elicited in gemcitabine resistance.

We further overexpressed *AKT1* and *GSK* plasmids in cells to observe their effects on *Gli-luc* activation. As shown in Fig 2, overexpression of AKT increased phospho-AKT^Ser473^, phospho-GSK3β^Ser9^, Gli2 expression, and *Gli-luc* activity, respectively (Fig 2D, upper and bottom). In the absence of the HH ligands, Gli proteins can be phosphorylated by GSK3β kinase to become repressor forms to inhibit the HH pathway [15]. GSK3 is a multifunctional serine/threonine kinase, which plays important roles in many activities, including embryonic development, glycogen metabolism, neuronal function, and cancer [24]. In particular, it is also involved in the resistance to chemo-, radio-, and targeted therapy of many cancers [25, 26], and might be a potential target for therapeutic intervention. However, its oncogenic or tumor-suppressive roles remain controversial [27]. GSK3β can be phosphorylated by AKT at its Ser9 residue to inactivate kinase function [28]. Therefore, *Flag-GSK*^*S9D*^ (constitutively inactive GSK3β) and *Flag-GSK*^*S9A*^ (constitutively active GSK3β) plasmids were used to elucidate their effects on the regulation of the Gli2 activation. As shown in Fig 2, overexpression of *Flag-GSK*^*S9D*^ in NTUB1 cells enhanced Gli2 expression and *Gli-luc* activity, respectively (Fig 2E, upper and bottom). Conversely, overexpression of *Flag-GSK*^*S9A*^ in NGR cells suppressed Gli2 expression and *Gli-luc* activity, respectively (Fig 2F, upper and bottom). Taken together, these results indicate that gemcitabine resistance occurs in UC cells partially via AKT/GSK3β–regulated non-canonical Gli2 activation.

### Involvement of AKT/Gli2 expression in resistance-related migration/invasion abilities

**Fig 3:**
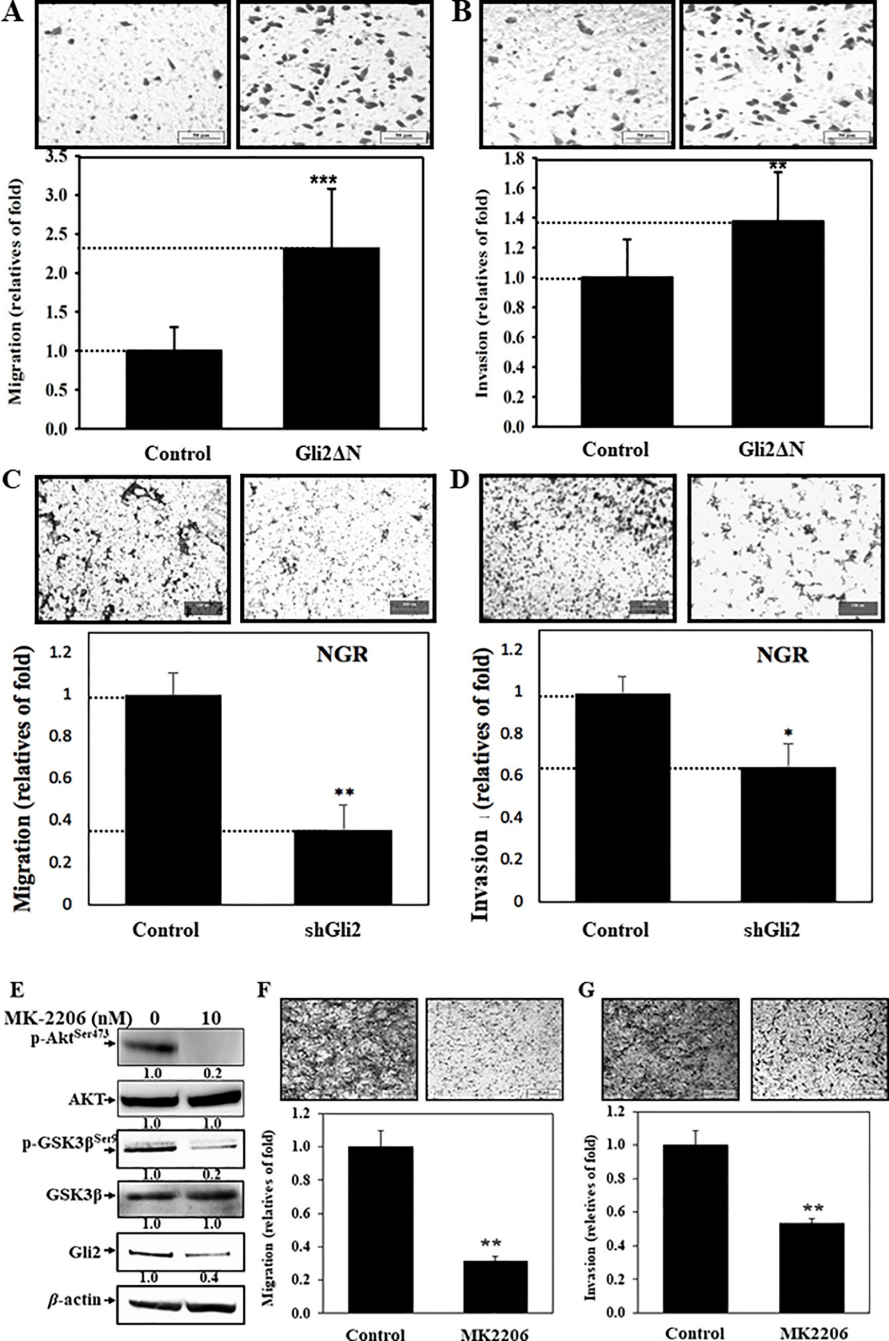
Involvement of Gli2 in cellular migration/invasion abilities. The (A, B) *pCS2MT-Gli2ΔN*, (C, D) Gli2 specific shRNA plasmids, and were transfected in cells for 24 h, then the abilities of migration and invasion were detected as described in the “Materials and Methods” section. (E-G) NGR cells were treated with specific phospho-AKT inhibitor MK2206 for 24 h. The phopho-AKT^ser473^, phospho-GSK3β^ser9^ and Gli2 protein were detected, and migration/invasion abilities were measured.

As shown in our recent results, we demonstrated that NGR exhibited higher invasive ability [29]. Herein, the effects of Gli2 on the events were addressed. Overexpression of *Gli2ΔN* in NTUB1 cells increased *n-cadherin*, *mmp2* expression, but decreased *e-cadherin* expression (data not shown), as well as promoted migration/invasion abilities of the cells (Fig 3A and 3B). In addition, knockdown of Gli2 in NGR cells suppressed cellular migration/invasion abilities (Fig 3C and 3D). Furthermore, we treated specific phospho-AKT inhibitor MK2206 and observed the attenuation of Gli2 expression and cellular migration/invasion abilities in NGR cells (Fig 3E–3G), and in T24_GR cells (S2A and S2B Fig), respectively. Therefore, we suggest that AKT/Gli2 activation was involved in the resistance-related migration/invasion.

### Contribution of Gli2 expression to gemcitabine resistant UC cells

**Fig 4:**
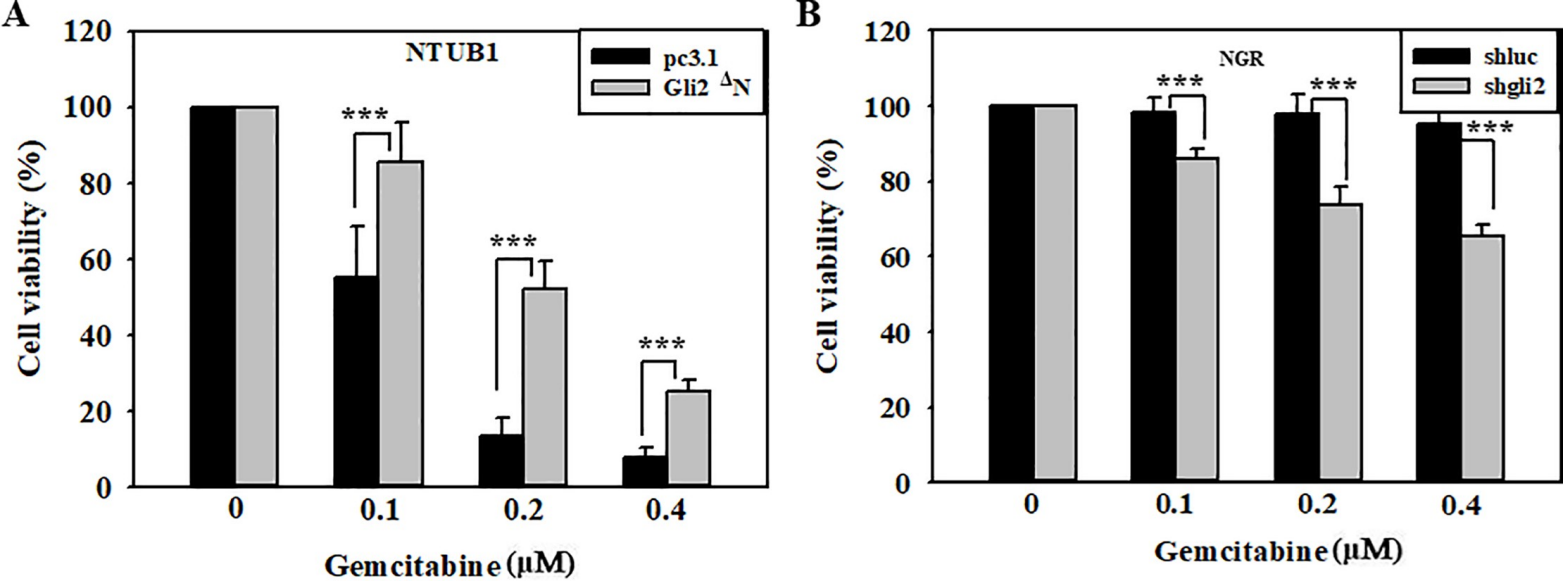
Contribution of Gli2 expression to gemcitabine resistant UC cells. (A) NTUB1 or (B) NGR cells were transfected with (A) *pCS2MT-Gli2ΔN* plasmids or (B) Gli2 specific shRNA plasmids, respectively, and then treated with various doses of gemcitabine (0−0.4 μM) for 72 h. Cells were trypsinized and counted numbers by using a counting chamber to measure cellular viability.

To further prove the involvement of Gli2 activation in the gemcitabine resistance, assessments of gain- and loss-of-function of Gli2 in UC cells were performed. As shown in Fig 4, overexpression of *Gli2ΔN* (active form of Gli2) in NTUB1 cells resulted in more resistance to gemcitabine (Fig 4A). However, knockdown of Gli2 by its specific shRNA led to increase cellular sensitivity to gemcitabine in NGR cells (Fig 4B), and in T24_GR cells (S3 Fig).

## Discussion

UC of the bladder is one of the critical malignancies in men worldwide. It is a chemo-sensitive disease; however, drug resistance and rapidly occurring relapse are the main reasons for treatment failure [8]. Therefore, understanding the drug-resistant mechanisms is needed to further improve chemotherapy efficacy. Many enzymes or specialized transporters are involved in gemcitabine metabolism, and any change might alter the sensitivity or even create resistance to gemcitabine [9]. For instance, high expression of hENT1 and dCK mRNA in resected specimens from patients with pancreatic cancer is associated with long overall survival, disease-free survival and disease progression [30]. Moreover, it has also been reported that deficiency of dCK enhances resistance to acquired gemcitabine [31], and transfection with hCNT3 greatly increases gemcitabine uptake to overcome resistance in pancreatic cancer [32].

In addition to the metabolic enzymes and transporters, many pathways have been proven to be critical to gemcitabine resistance. For example, activation of the PI3K/AKT pathway is associated with gemcitabine resistance in breast cancer [33]. Accumulating evidence also indicates that HH, Wnt and Notch pathways become reactivated in gemcitabine-resistant pancreatic cancer [34]. However, whether the HH pathway becomes reactivated in gemcitabine-resistant UC has not yet been proven.

**Fig 5:**
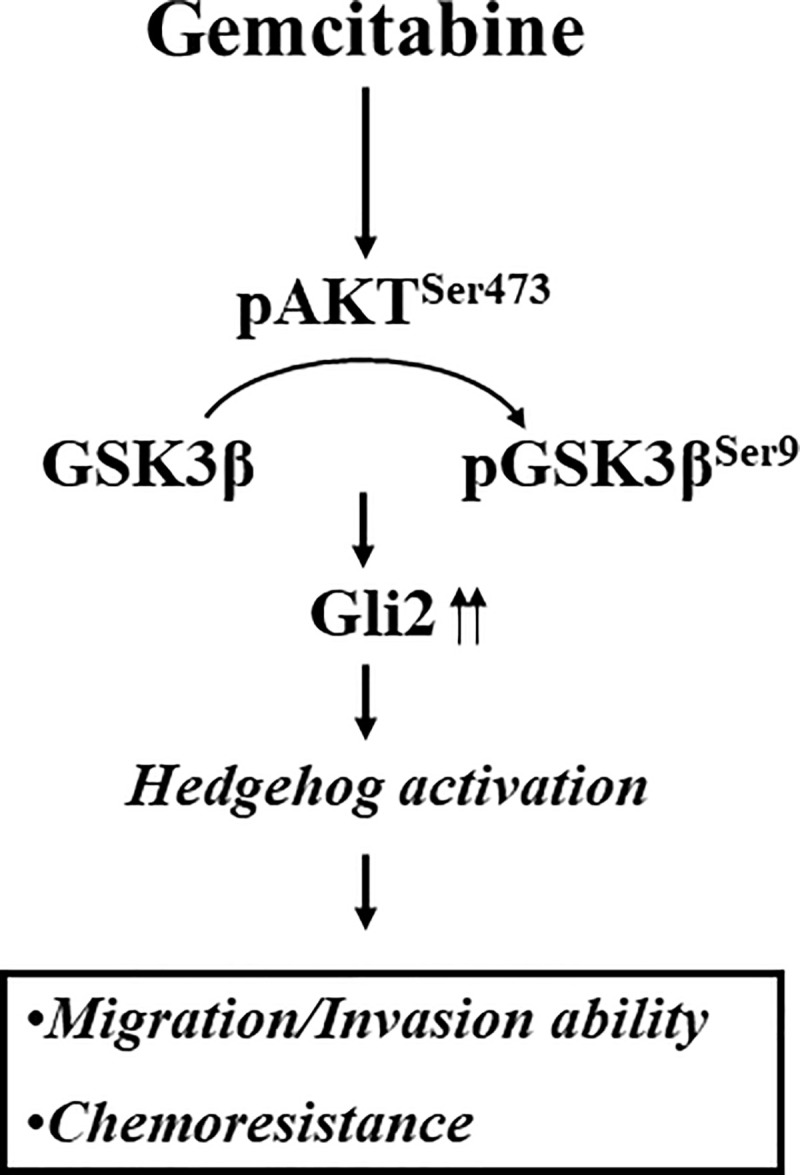
Scheme illustrates the involvement of Gli2-dependent pathway in gemcitabine resistant UC cells. The possible mechanisms identified from the *in vitro* results of present study were summarized in this cartoon graph. Besides the involvement of aberrant gemcitabine metabolism, gemcitabine can activate AKT^Ser473^ phosphorylation to inactivate GSK3β kinase by phosphorylating its Ser9 residue. The inactivated GSK3β leads to stabilize Gli2 proteins to induce its downstream target genes expressions, which promotes migration/invasion abilities and resistance to gemcitabine.

In the present work, novel mechanisms of gemcitabine resistant UC were provided as follows. First, non-canonical Gli2-dependent HH pathway mediates gemcitabine-resistant mechanisms. Second, gemcitabine-induced AKT/GSK3β pathway contributes to Gli2-dependent HH pathway (Fig 5). As described above, non-canonical HH pathway is another type of pathway associated with HH pathway components that bypasses the requirement for the HH ligands, but also plays critical roles in tumorigenesis and drug resistance [16]. Notably, such a situation might profoundly affect the tumor cells as well as the stromal cells, and challenge the efficacy of several HH inhibitors in clinical trials of many solid tumors [16]. Increasing evidence has been reported that several pathways are involved in the non-canonical HH pathway, such as the Ras/MEK/ERK, PI3K/AKT, EGFR, NF-κB, TGFβ, and Wnt pathways [16, 35, 36]. These pathways can interact with the HH pathway to contribute to tumor growth, metastasis, and drug-resistance, and then provide opportunities for combination therapies in cancer [16]. In particular, the PI3K/AKT pathway is associated with the important functions of growth, proliferation, and survival, which might lead to the cancer cells resisting chemotherapy [37]. The oxidative stress can also induce the NF-kB pathway, which is another downstream effector in the AKT pathway [38], and is related to gemcitabine resistance in pancreatic cancer and UC [39, 40]. Recently, we also had demonstrated that gemcitabine-induced TGIF expression can activate the PI3K/AKT pathway and MMPs cascades to enhance the aggressiveness and chemo-resistance in UC cells [29].

In the aberrant HH pathway, the activated Gli protein translocates into nucleus to induce several genes’ expression, such as Gli1, receptor PTCH, insulin-like growth factor-binding protein, Bcl2, cyclin D2, SNAIL, and osteopontin (OPN) [41, 42]. Tang et al. reported that Gli1 and Gli2 are required for EMT in human trophoblasts [43], which indicates that the AKT/GSK3β pathway might be through the HH pathway, regulating EMT to promote metastasis and resistance to gemcitabine. Accumulating evidence also indicates that HH pathway can regulate some stem cell markers to induce cancer stem cell formation, including bladder cancer [13, 44], thus conferring resistance to gemcitabine [34]. In our results, we observed that gemcitabine-resistant UC cells expressed higher Gli2 activation (Figs 1 and 2), and sphere formation (data not shown). Overexpression of Gli2 in cells could also increase resistance to gemcitabine treatment (Fig 4A). However, we only observed partial reduction of resistance to gemcitabine after knockdown of Gli2 in NGR and T24_GR cells (Fig 4B and S3 Fig). The reason might be due to the modest transfection efficiency, or due to the gemcitabine-resistant cells already bearing irreversible stem cell properties, and then leading to the results as described above (Fig 4B and S3 Fig).

In addition, OPN is a secreted phosphoglycoprotein; abundant in the tumor microenvironment, it promotes proinflammatory conditions, and then enhances tumor progression and metastasis. Inflammation is one of the cancer hallmarks that create the tumor microenvironment to affect tumorigenesis, metastasis, or drug-resistance [45]. Recently, it has been reported that OPN can non-classically induce AKT phosphorylation to inactivate GSK3β, and then elicit Gli-mediated transcription [26]. Whether OPN is involved in the gemcitabine-activated HH pathway in resistant UC to cross-talk with other cells in tumor microenvironment will be explored.

Recently, combination therapy with gemcitabine plus HH inhibitor to treat gemcitabine-resistant pancreatic cancer has been reported [34]. Ormeloxifene, a non-hormonal, nonsteroidal oral contraceptive molecule, can suppress HH-induced desmoplasia to enhance cellular sensitivity to gemcitabine [46]. Cyclopamine, an HH pathway inhibitor, can inhibit the HH pathway to reverse gemcitabine resistance in pancreatic cancer [47]. Several clinical trials of HH pathway inhibitor in combination with gemcitabine in pancreatic cancer are currently ongoing (http://www.clinicaltrials.gov/). However, some clinical trials have been reported that the combination treatment with SMO inhibitor and gemcitabine in patients with pancreatic cancer was not better than gemcitabine alone [48–50]. We also demonstrated that treatment with SMO inhibitor GDC0449 in NGR cells only partially inhibited Gli2 activation (Fig 2C). According to our results, we suggest that AKT-dependent non-canonical Gli2 pathway also plays a critical role in the gemcitabine resistance, however, these *in vitro* results have limitations and need to be strengthened by the preclinical studies to evaluate their applications in UC patients.

## Supporting information

S1 Fig The off-target effects of shGli2. (A) Cells were co-transfected with reporters and *pCS2MT-Gli2ΔN* plasmids in T24 cells, and then harvested for the detection of their *Gli2-luc* activity. (B) To reduce the off-target effects, we co-transfected 4 specific shRNAs with different targets to Gli2 in NGR cells. The pLKO.1-shLuc vector was used as a vehicle control. (C) To perform rescue experiments, we co-transfected reporters, *shGli2*, and *pCS2MT-Gli2ΔN* plasmids in NGR cells, and then harvested for the detection of their *Gli2-luc* activity.
(TIF)

S2 Fig Involvement of AKT activation in cellular migration/invasion abilities. T24_GR cells were treated with specific phospho-AKT inhibitor MK2206 for 24 h. (A) The phopho-AKT^ser473^, phospho-GSK3β^ser9^ and Gli2 protein were detected, and (B) cellular migration abilities were measured.
(TIF)

S3 Fig Contribution of Gli2 expression to gemcitabine resistant UC cells. T24_GR cells were transfected with *shGli2* plasmids, and then treated with various doses of gemcitabine (0−0.4 μM) for 72 h. Cells were trypsinized and counted numbers by using a counting chamber to measure cellular viability.
(TIF)

S1 Raw images (PDF)
