# Supplementary figures and images for: Gemcitabine-induced Gli-dependent activation of hedgehog pathway resists to the treatment of urothelial carcinoma cells
Source: PLoS One. 2021 Jul 8;16(7):e0254011. doi: 10.1371/journal.pone.0254011 (PMC8266077; doi:10.1371/journal.pone.0254011)

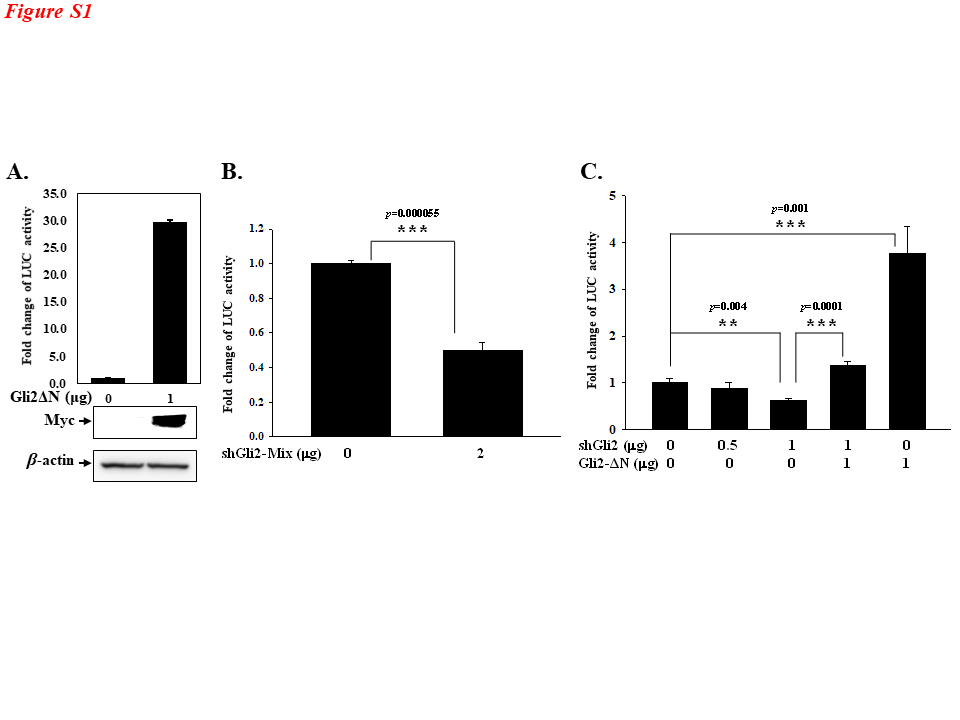

Supplement: S1 Fig — (A) Cells were co-transfected with reporters and pCS2MT-Gli2ΔN plasmids in T24 cells, and then harvested for the detection of their Gli2-luc activity. (B) To reduce the off-target effects, we co-transfected 4 specific shRNAs with different targets to Gli2 in NGR cells. The pLKO.1-shLuc vector was used as a vehicle control. (C) To perform rescue experiments, we co-transfected reporters, shGli2, and pCS2MT-Gli2ΔN plasmids in NGR cells, and then harvested for the detection of their Gli2-luc activity. (TIF) [file pone.0254011.s001.tif]

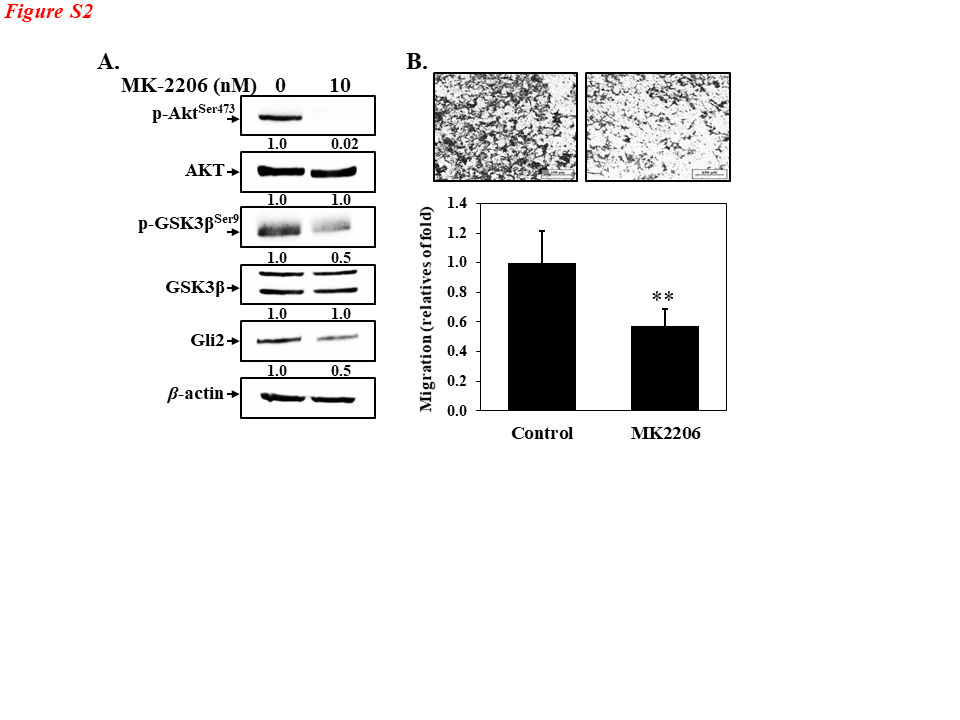

Supplement: S2 Fig — T24_GR cells were treated with specific phospho-AKT inhibitor MK2206 for 24 h. (A) The phopho-AKTser473, phospho-GSK3βser9 and Gli2 protein were detected, and (B) cellular migration abilities were measured. (TIF) [file pone.0254011.s002.tif]

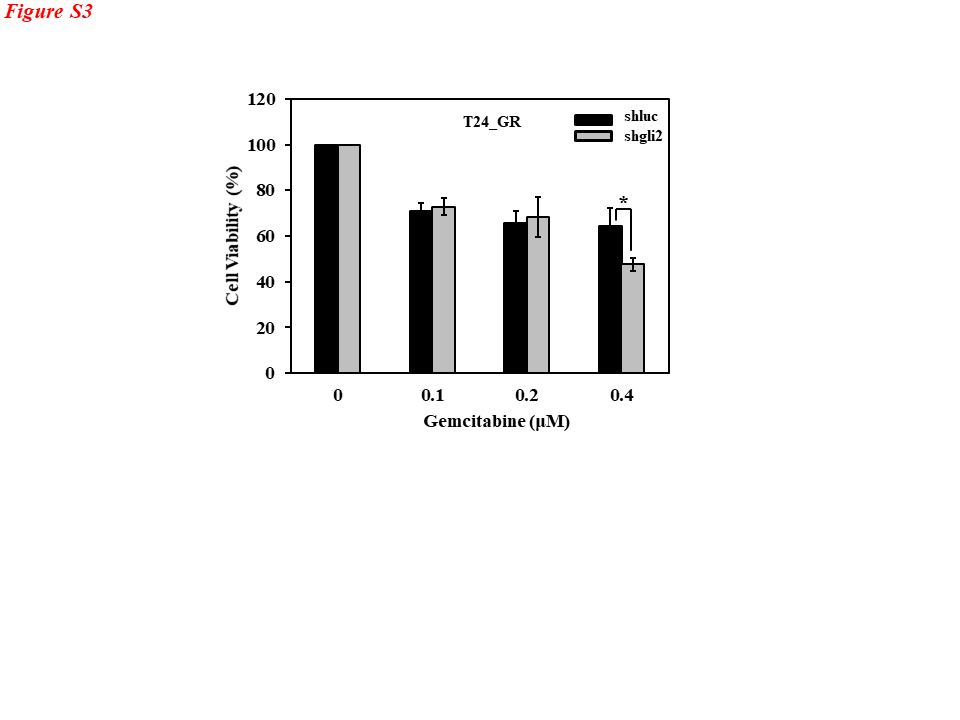

Supplement: S3 Fig — T24_GR cells were transfected with shGli2 plasmids, and then treated with various doses of gemcitabine (0−0.4 μM) for 72 h. Cells were trypsinized and counted numbers by using a counting chamber to measure cellular viability. (TIF) [file pone.0254011.s003.tif]
