# Supplementary material for: Gemcitabine-induced Gli-dependent activation of hedgehog pathway resists to the treatment of urothelial carcinoma cells
Source: PLoS One. 2021 Jul 8;16(7):e0254011. doi: 10.1371/journal.pone.0254011 (PMC8266077; doi:10.1371/journal.pone.0254011)
Supplement: S1 Raw images — (PDF) [file pone.0254011.s004.pdf]

Figure 1E Anti-dCK

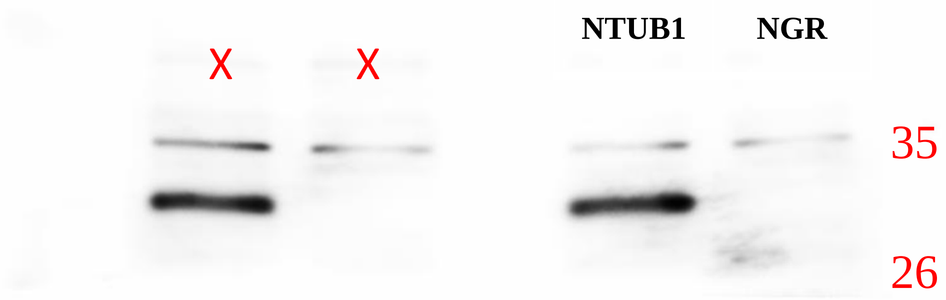

Figure 1E Anti-hENT1

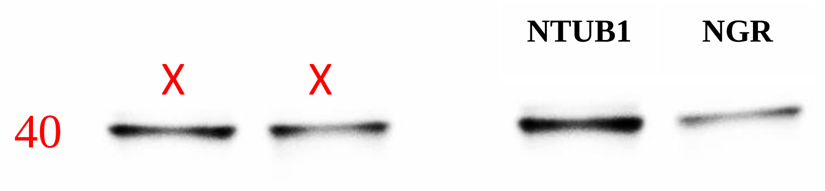

Figure 1E Anti-α-tubulin

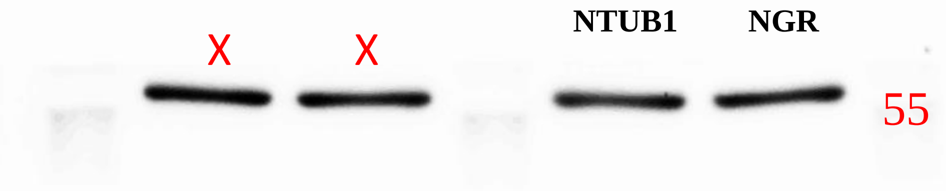

Figure 1F Anti-pAkt

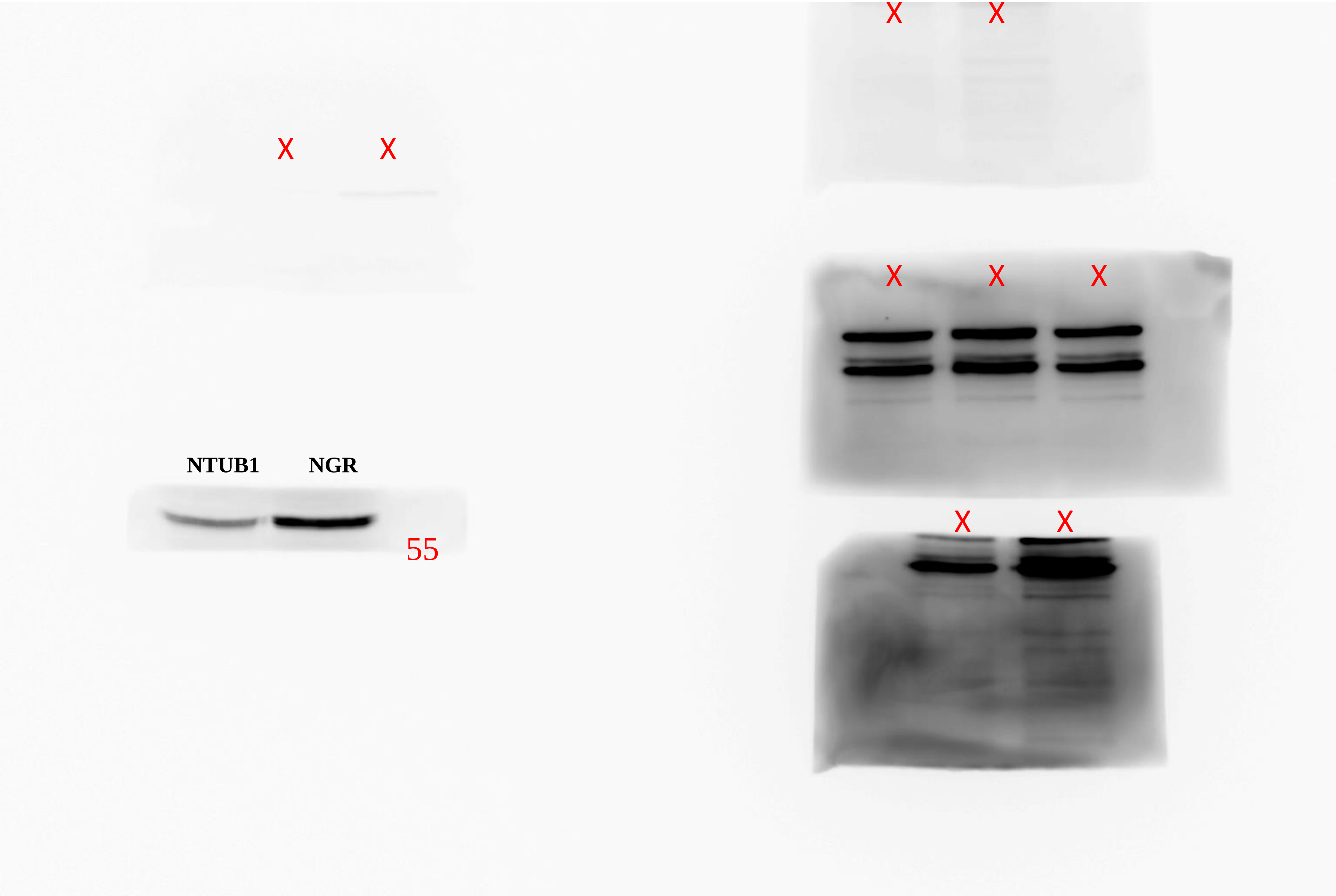

Figure 1F Anti-AKT

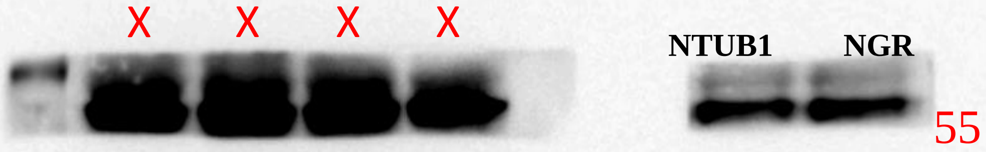

Figure 1F Anti-pGSK3β

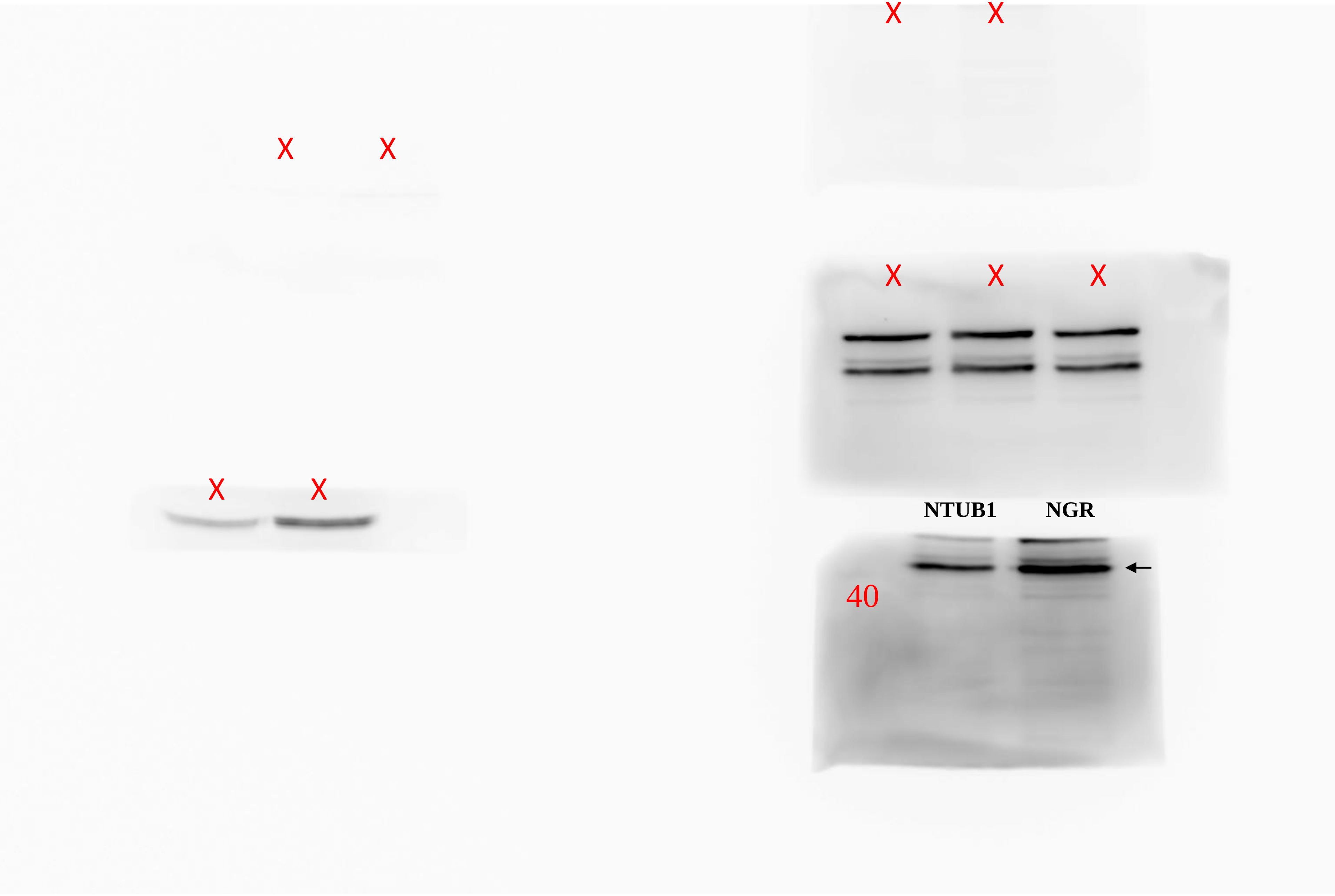

Figure 1F Anti-GSK3β

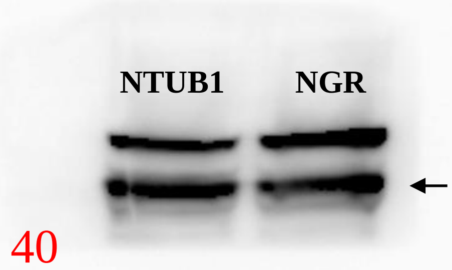

Figure 1F Anti-Gli2

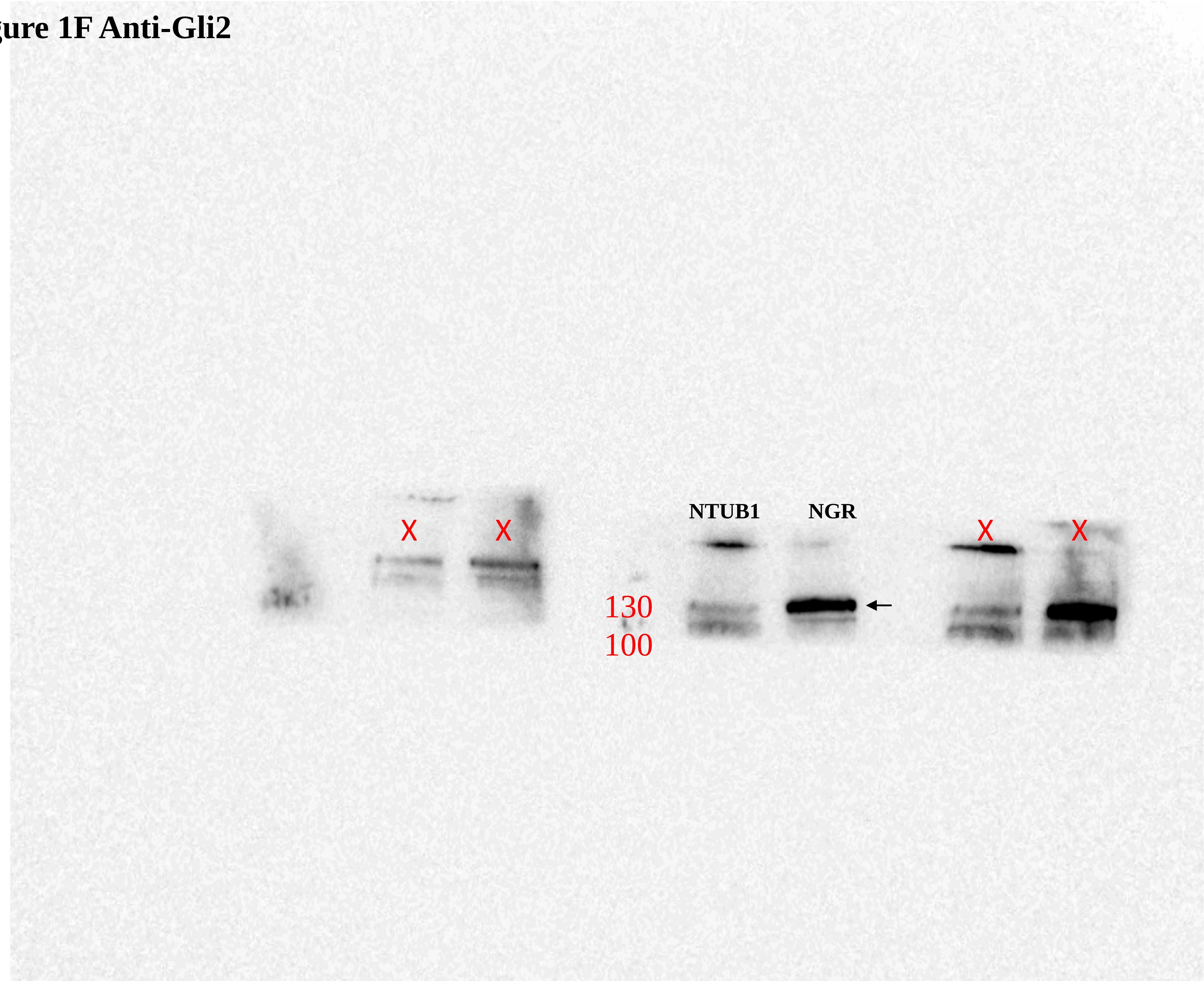

Figure 1F Anti-β-tubulin

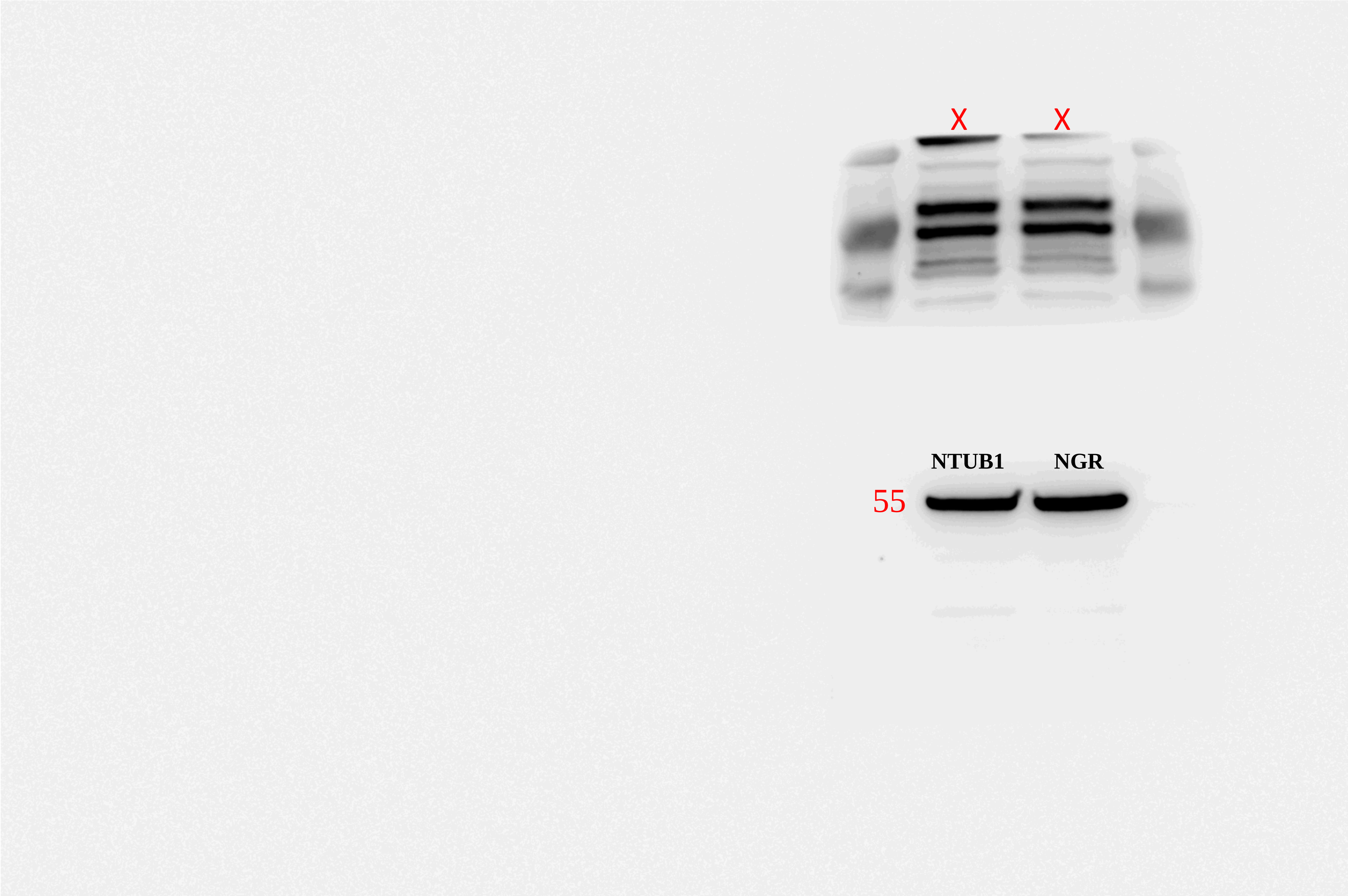

Figure 1G Anti-pAkt

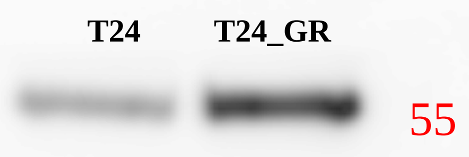

Figure 1G Anti-Akt

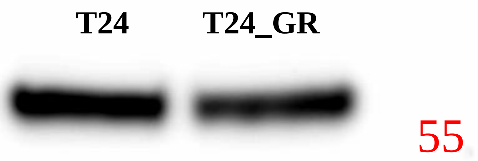

Figure 1G Anti-pGSK3β

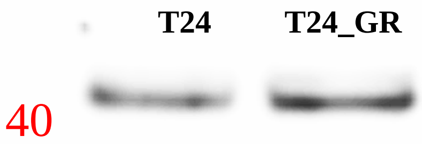

Figure 1G Anti-GSK3β

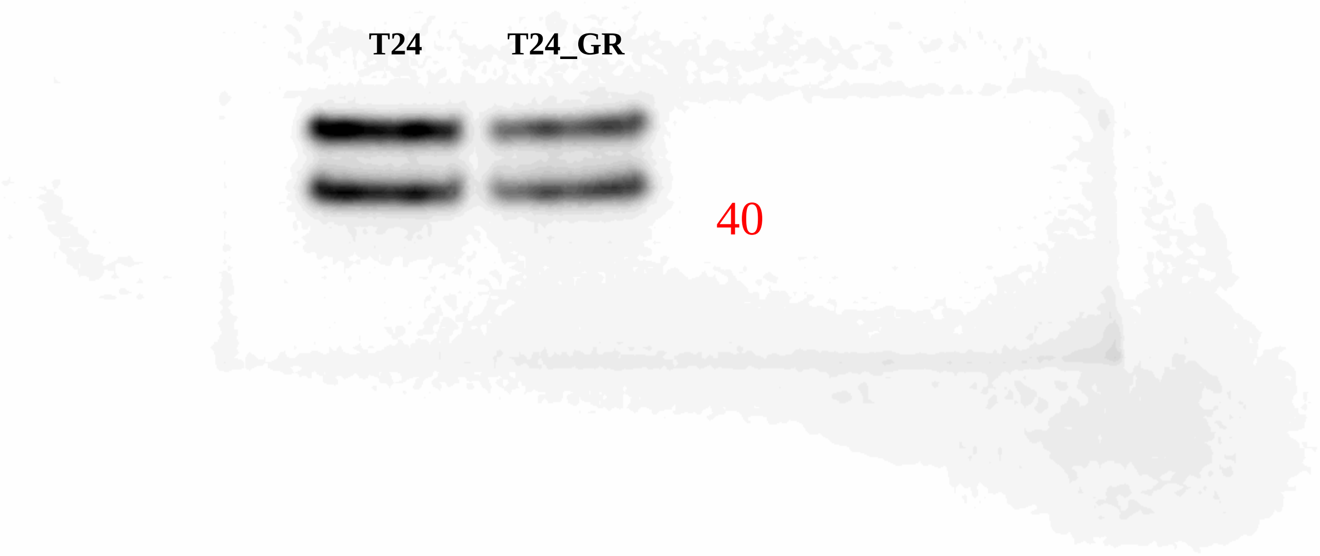

Figure 1G Anti-Gli2

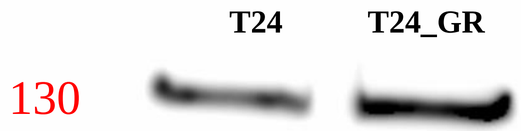

Figure 1G Anti-β-tubulin

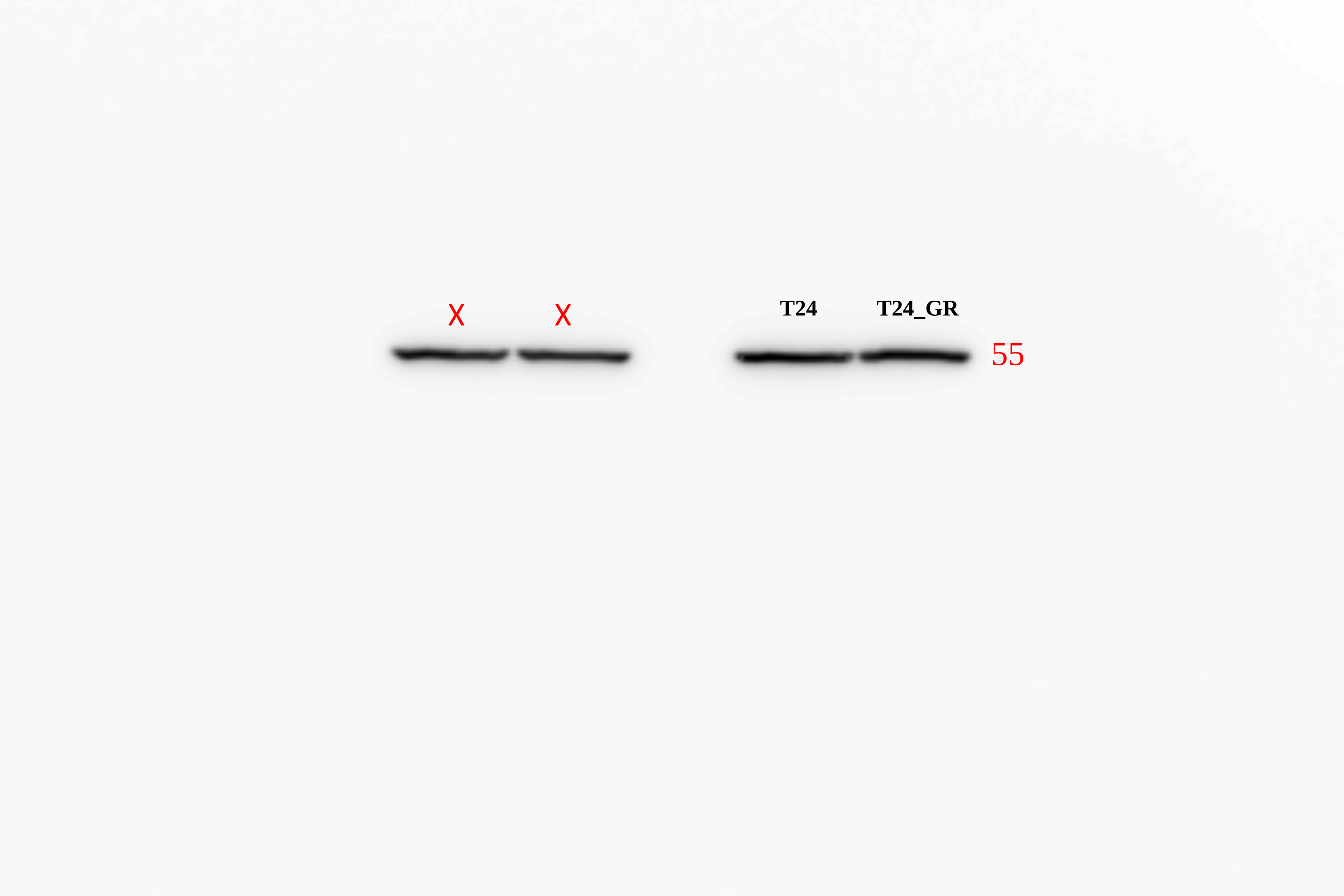

Figure 2A Anti-c-myc

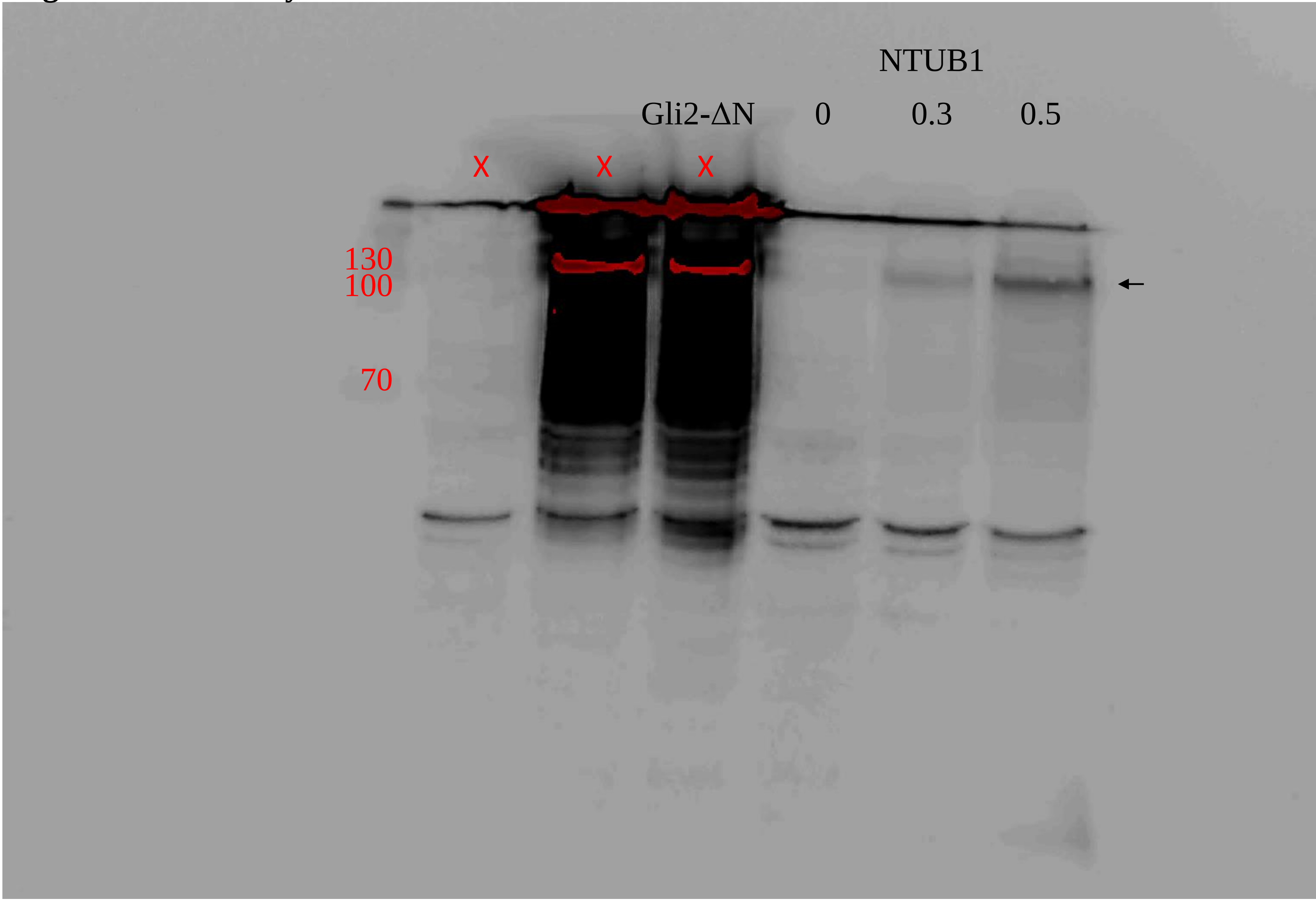

Figure 2B Anti-β-tubilin

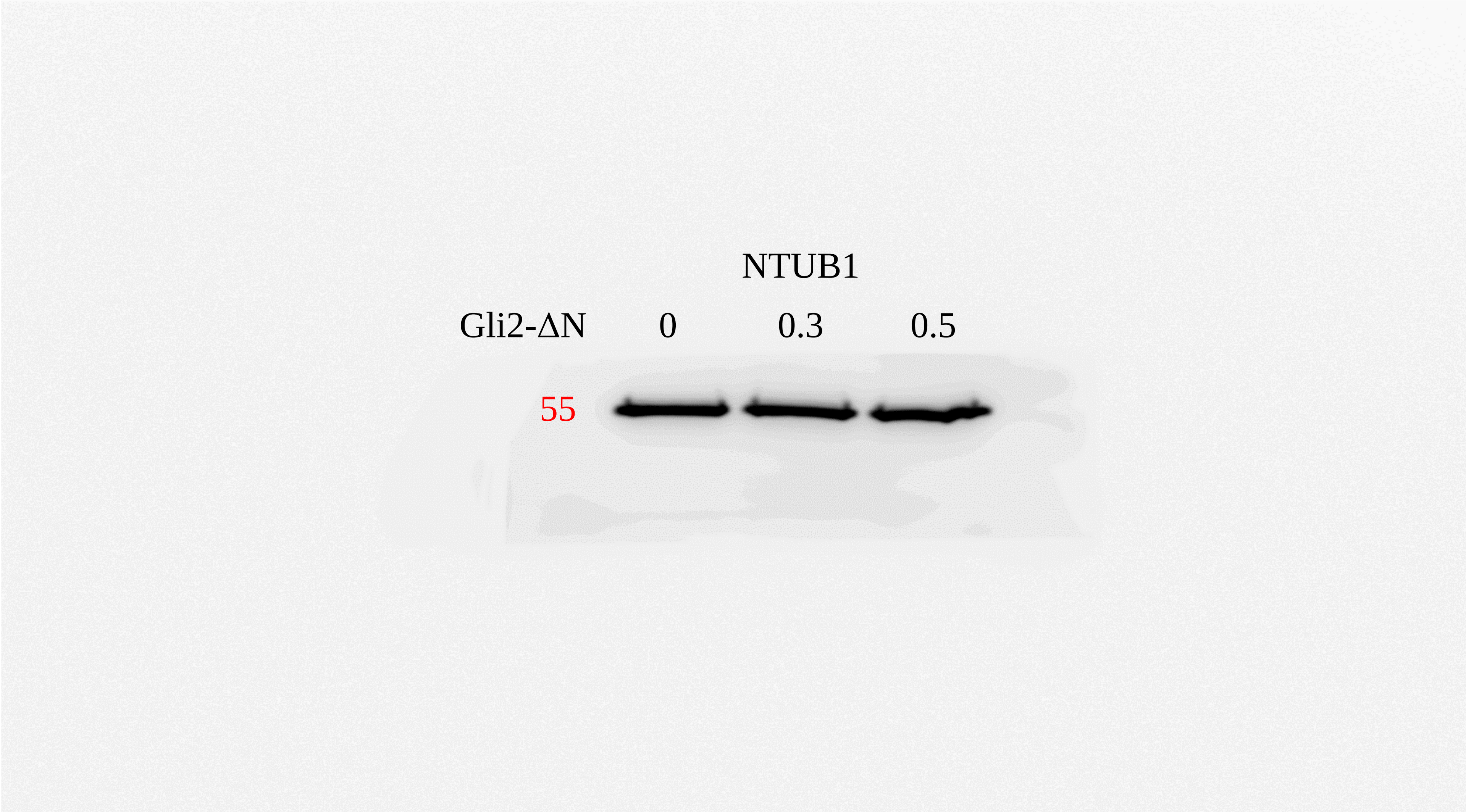

Figure 2B Anti-Gli2

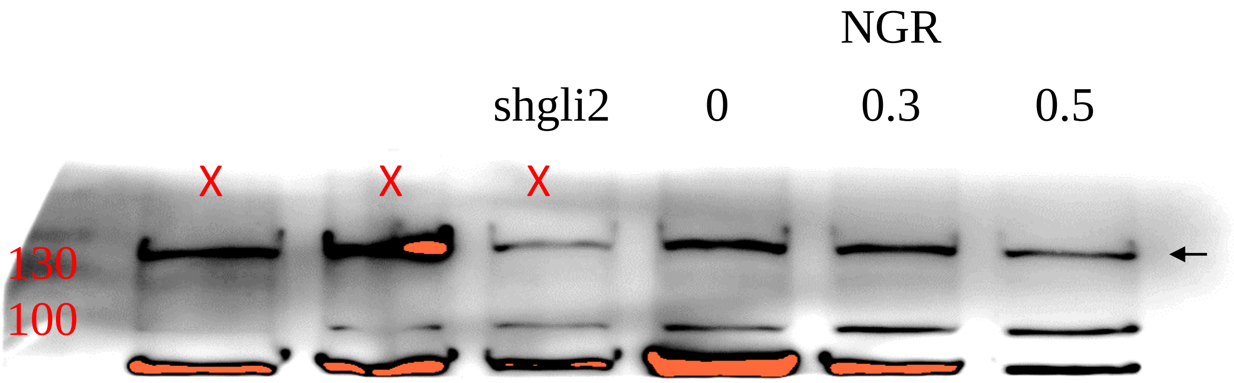

Figure 2C Anti-SMO

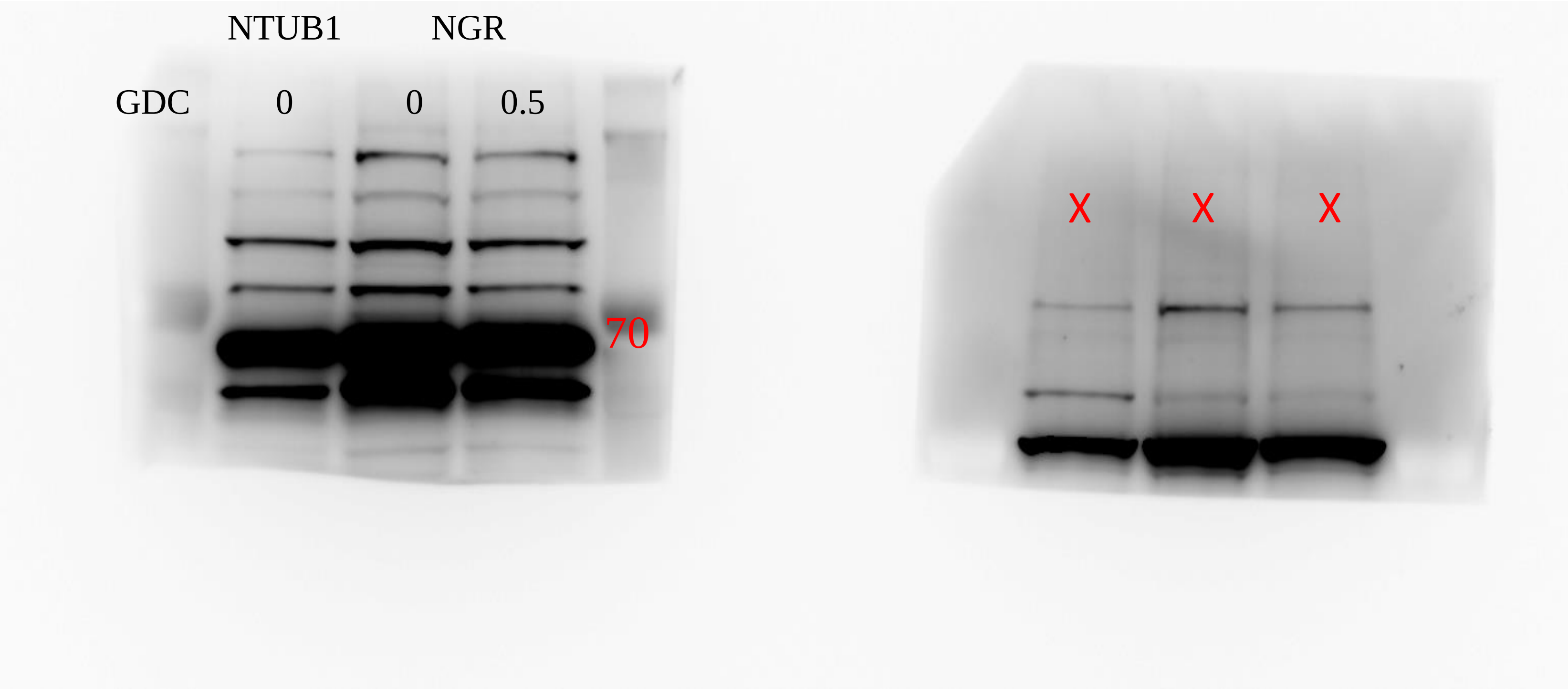

Figure 2C Anti-Gli2

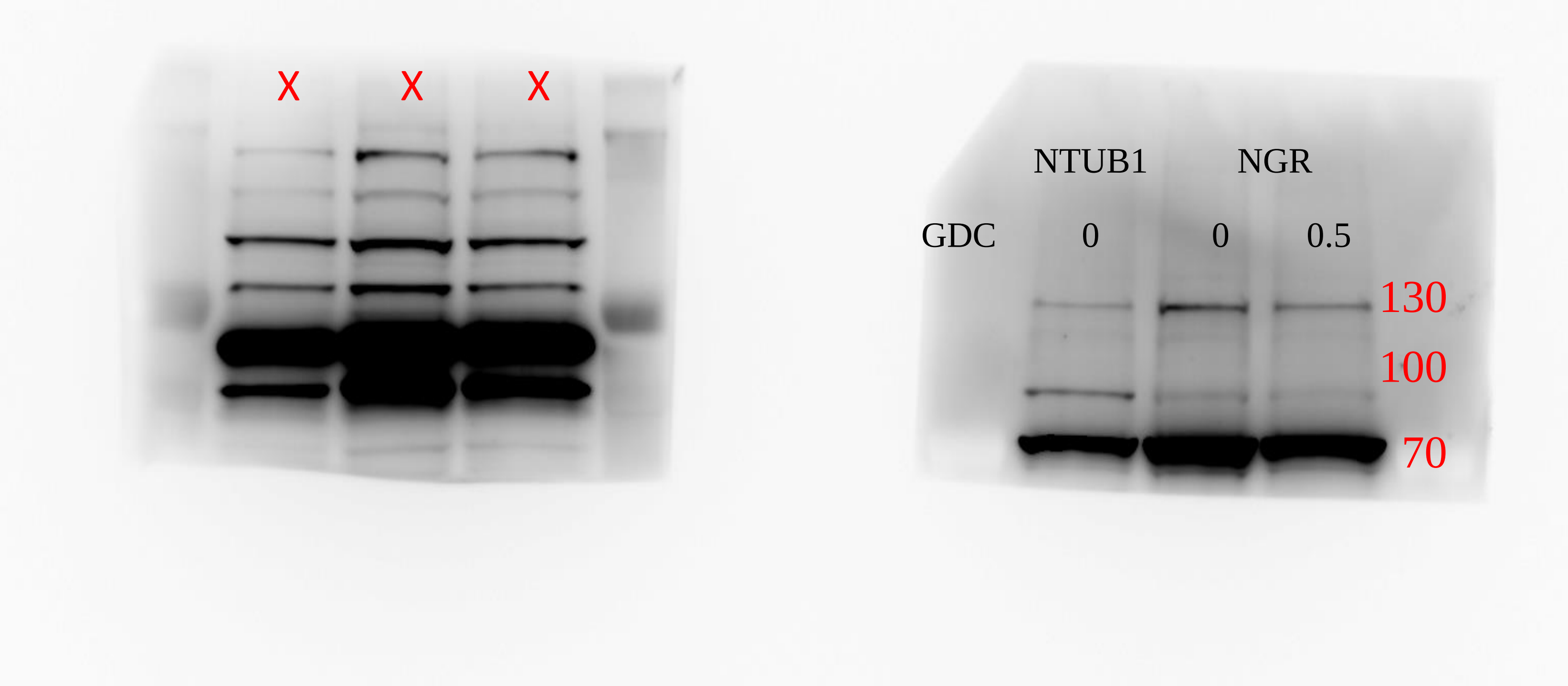

Figure 2C Anti-β-tubulin

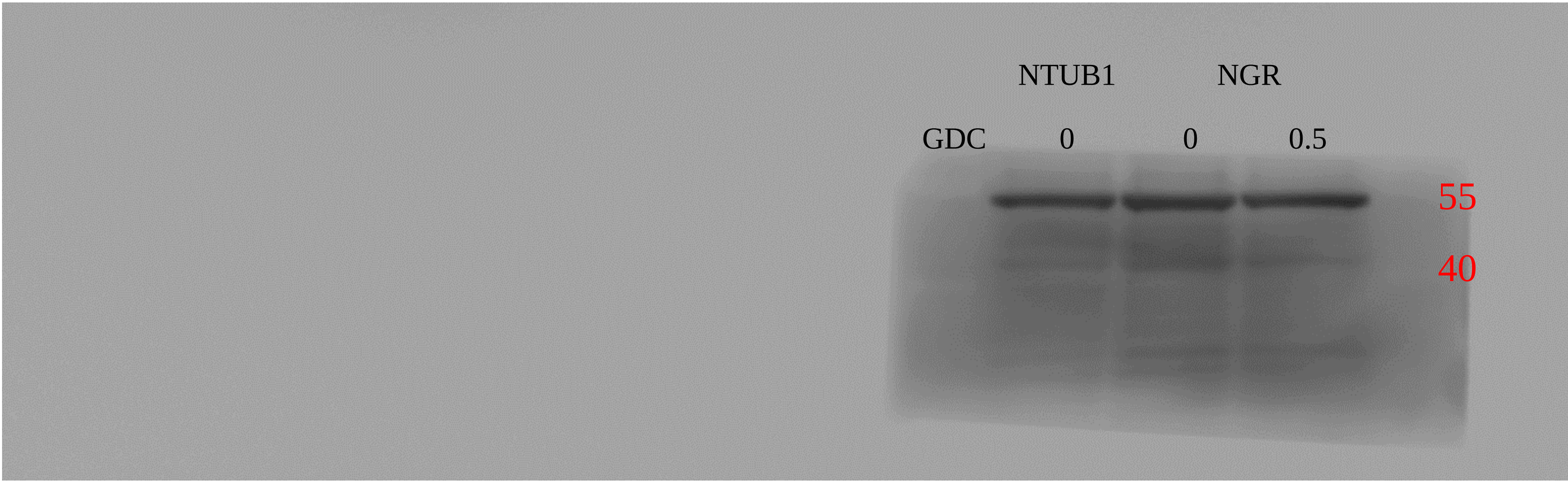

Figure 2D Anti-pAKT

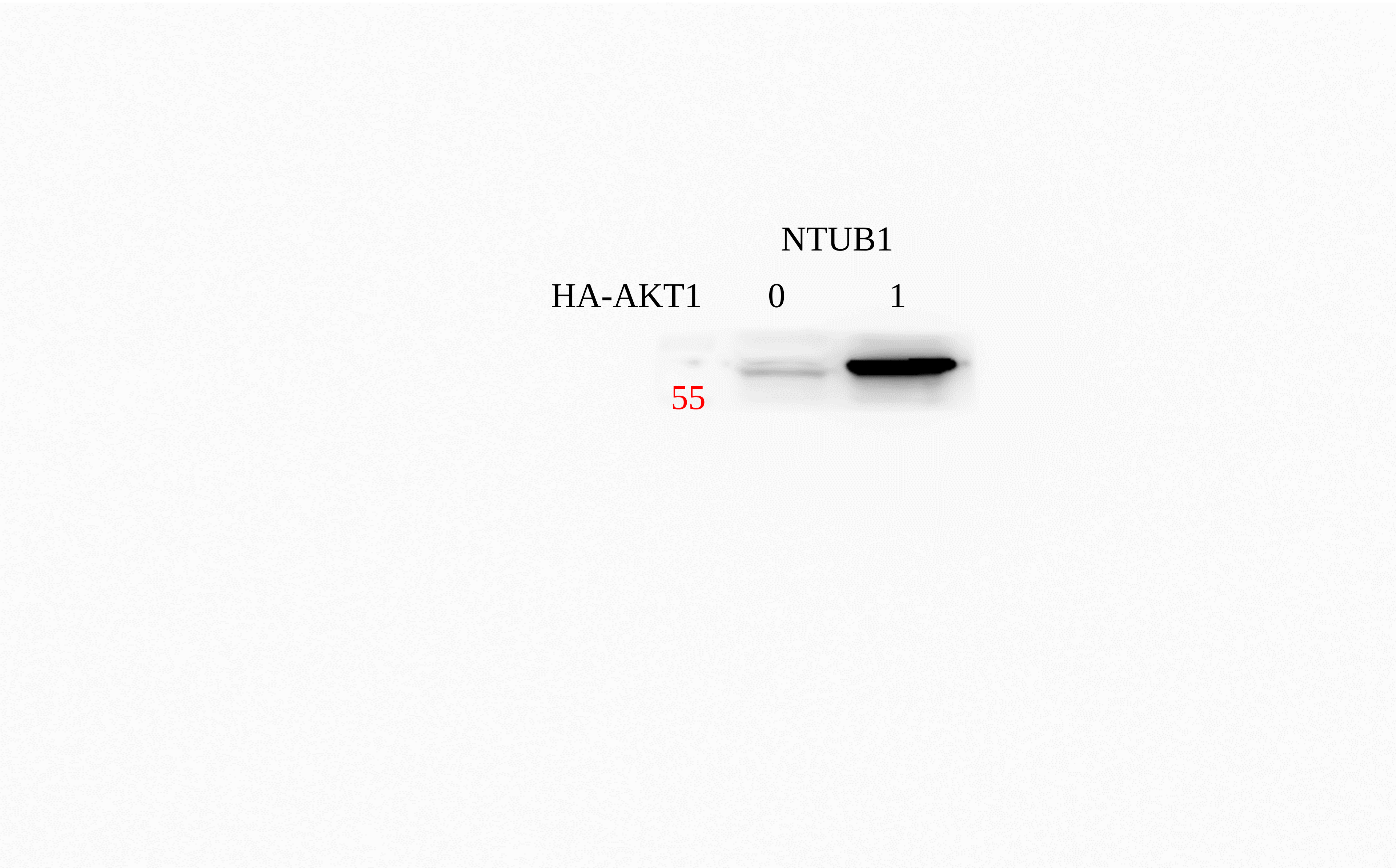

Figure 2D Anti-AKT

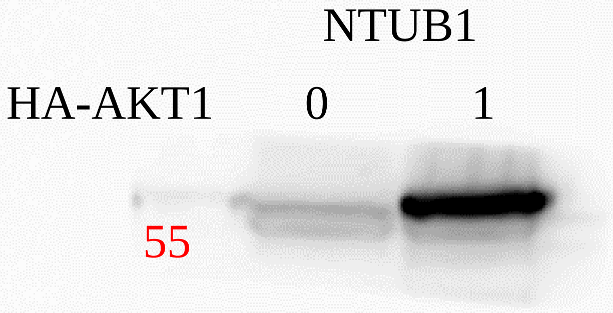

Figure 2D Anti-pGSK3β

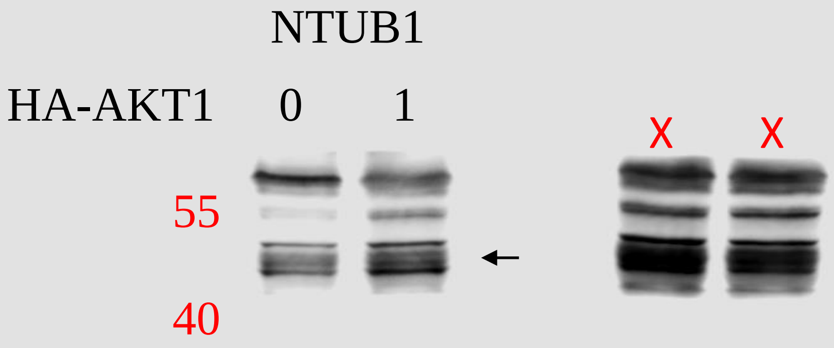

Figure 2D Anti-GSK3β

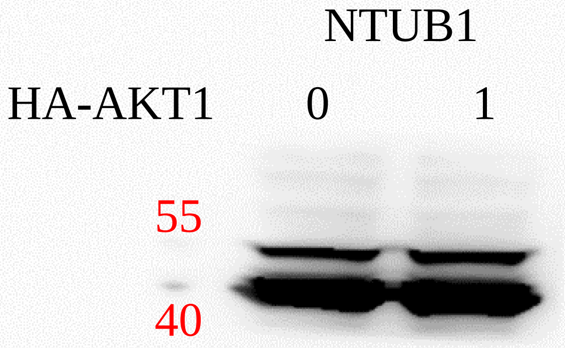

Figure 2D Anti-Gli2

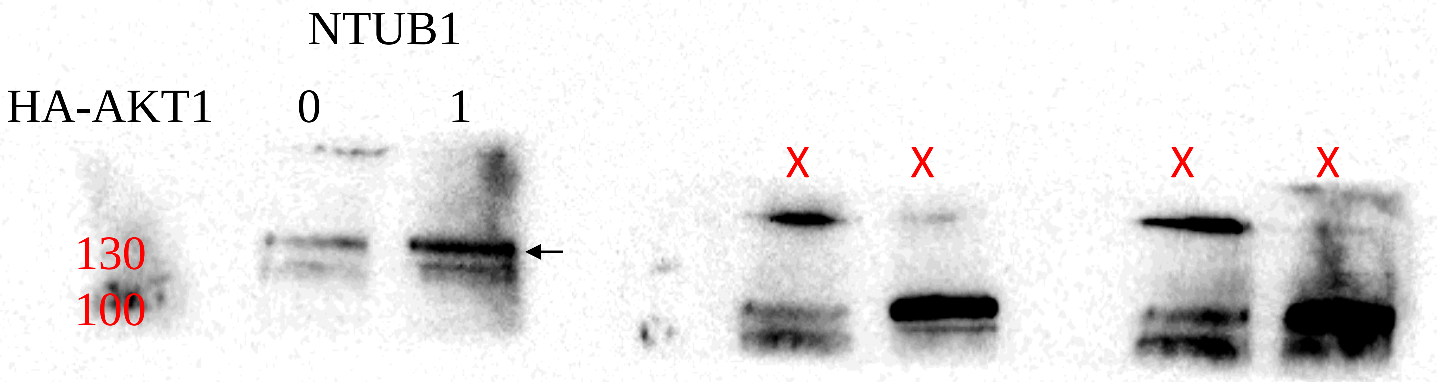

Figure 2D Anti-β-actin

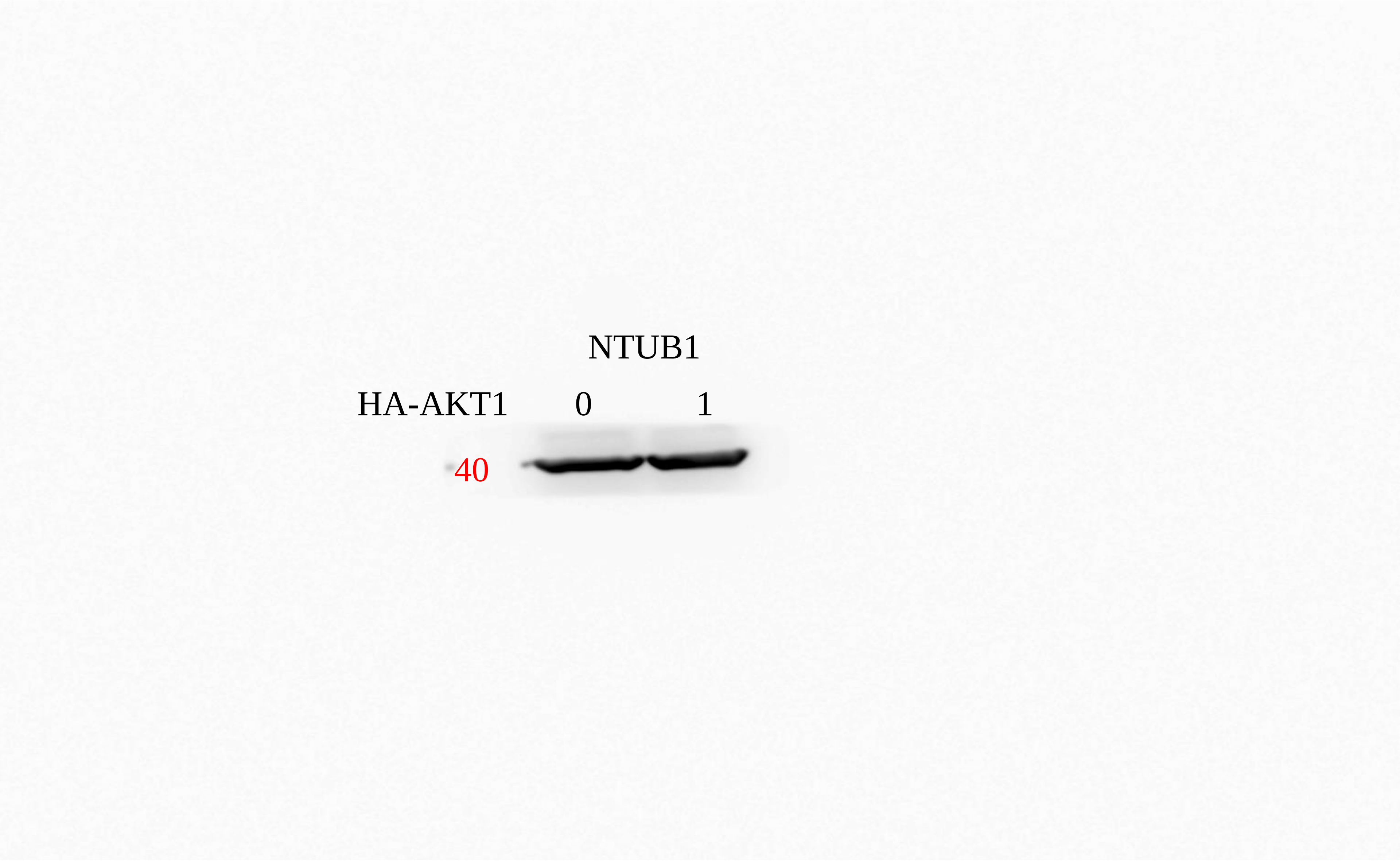

Figure 2E Anti-GSK3β

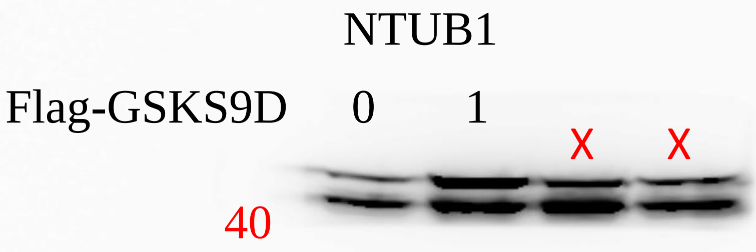

Figure 2E Anti-Flag

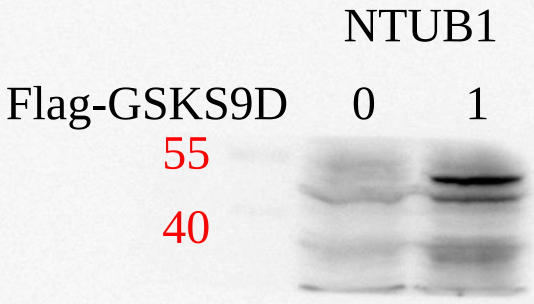

Figure 2E Anti-Gli2

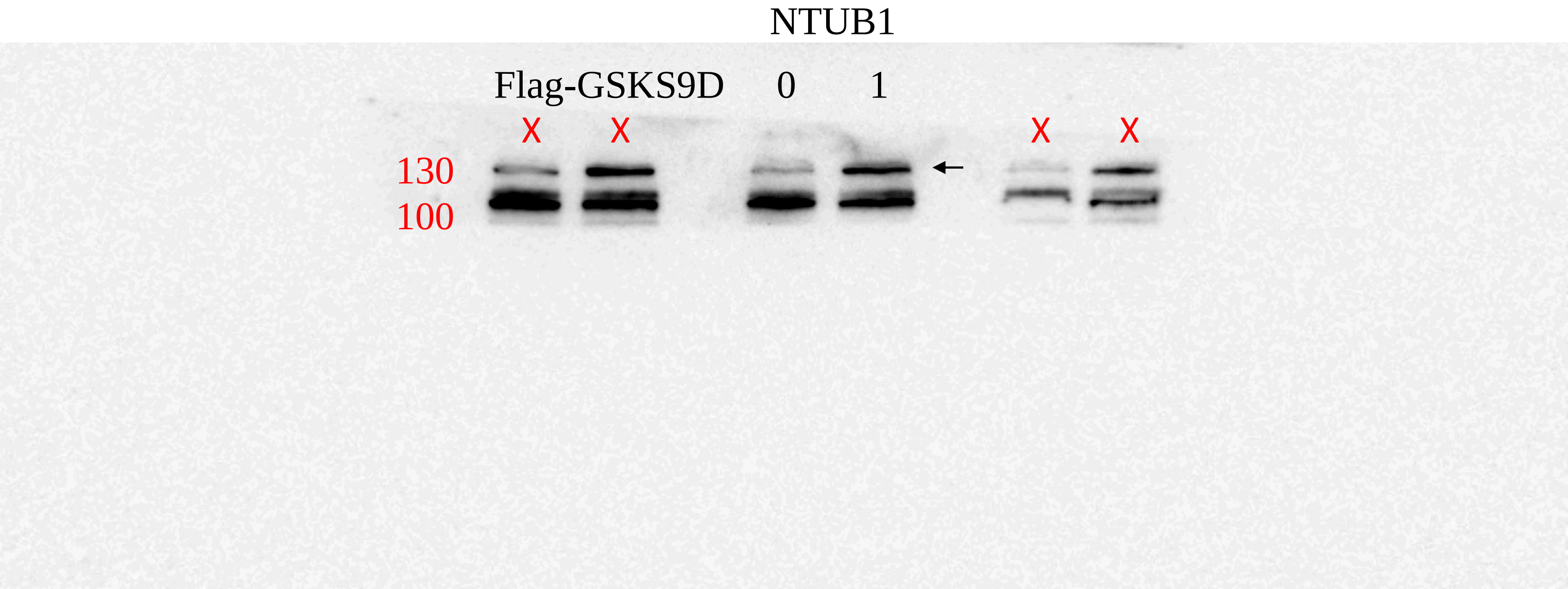

Figure 2E Anti-β-tubulin

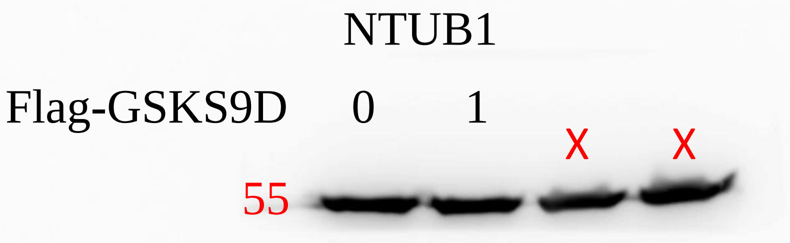

Figure 2F Anti-GSK3β

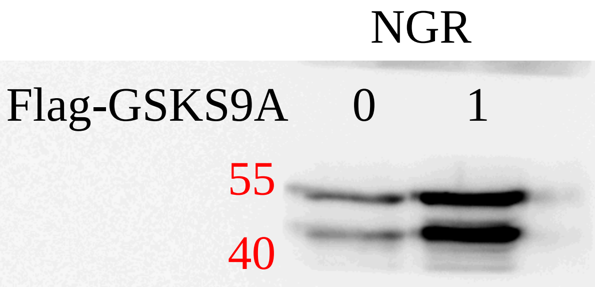

Figure 2F Anti-Flag

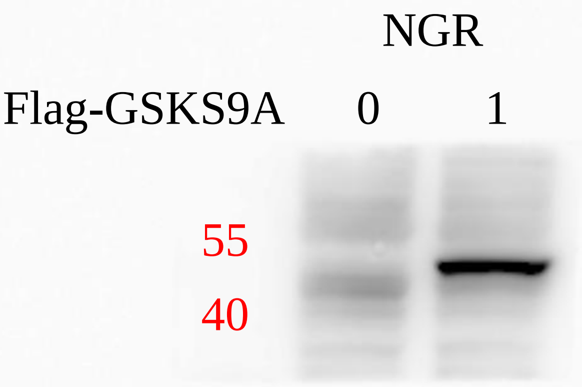

Figure 2F Anti-Gli2 無原圖

The original figure has been missed due to leaving of the person several years ago.

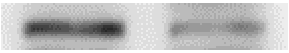

Figure 2F Anti-β-actin 無原圖

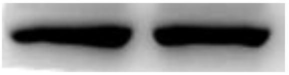

The original figure has been missed due to leaving of the person several years ago.

Figure 3E Anti-AKT

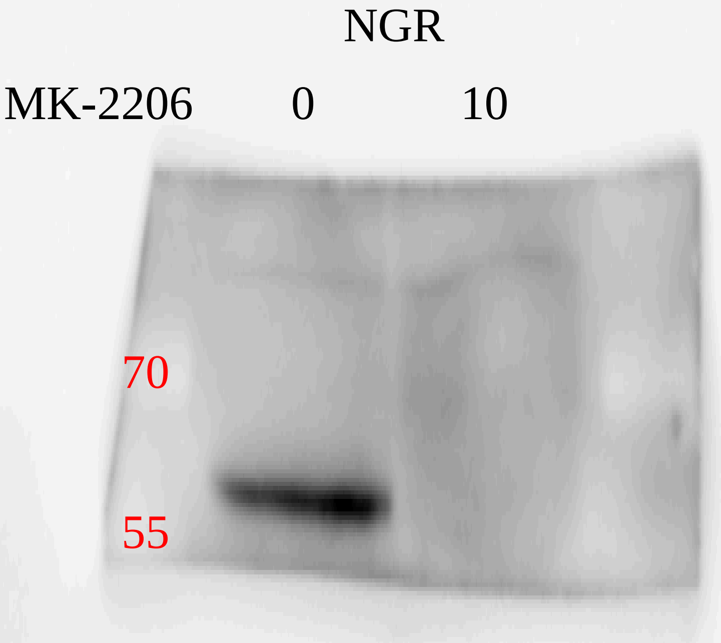

Figure 3E Anti-AKT

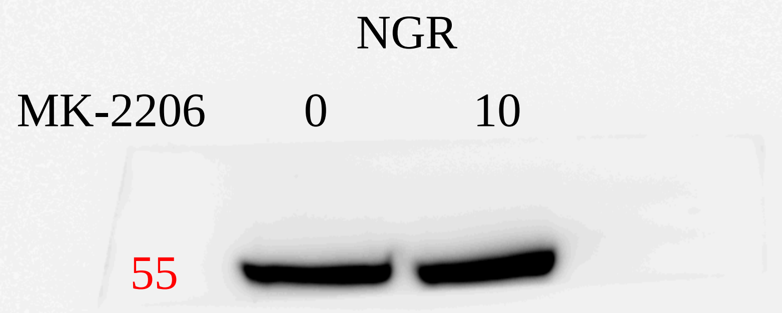

Figure 3E Anti-pGSK3β

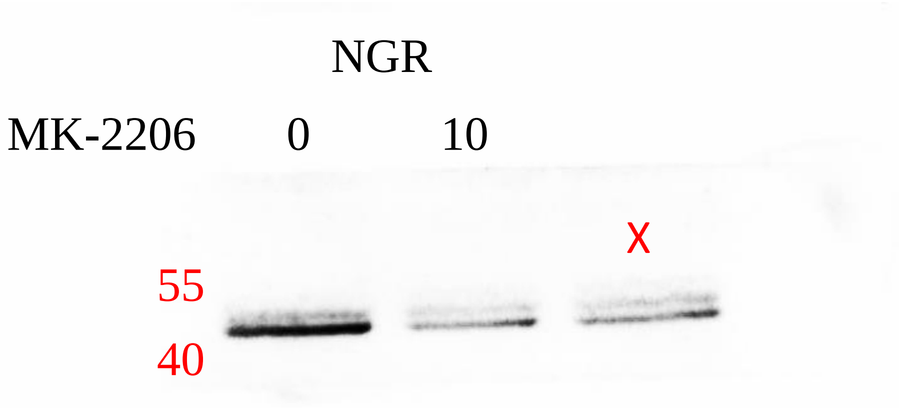

Figure 3E Anti-GSK3β

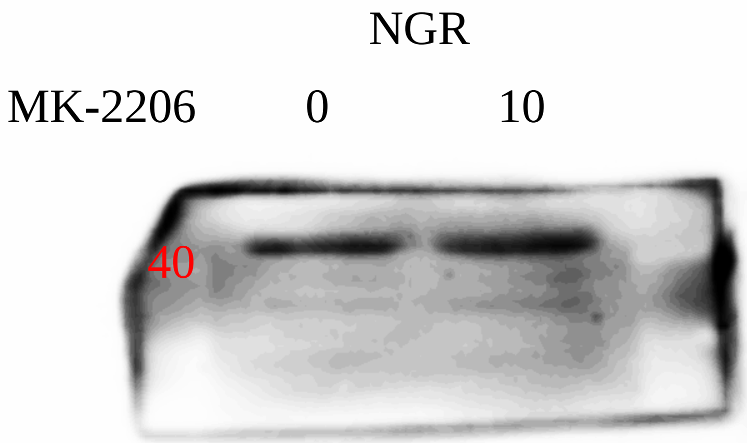

Figure 3E Anti-Gli2

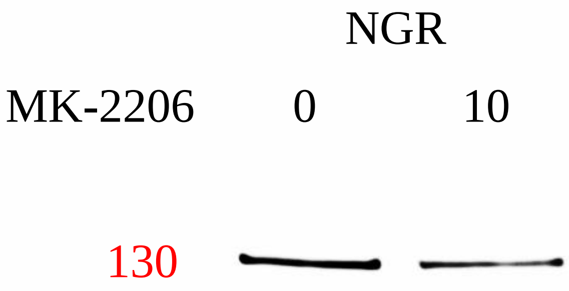

Figure 3E Anti-β-actin

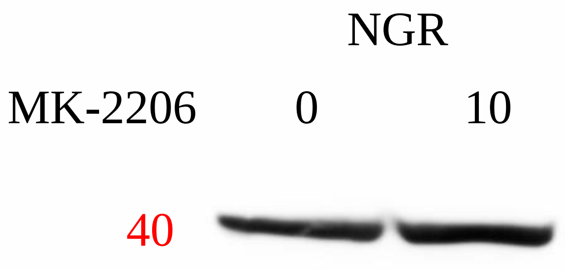

Figure S2A

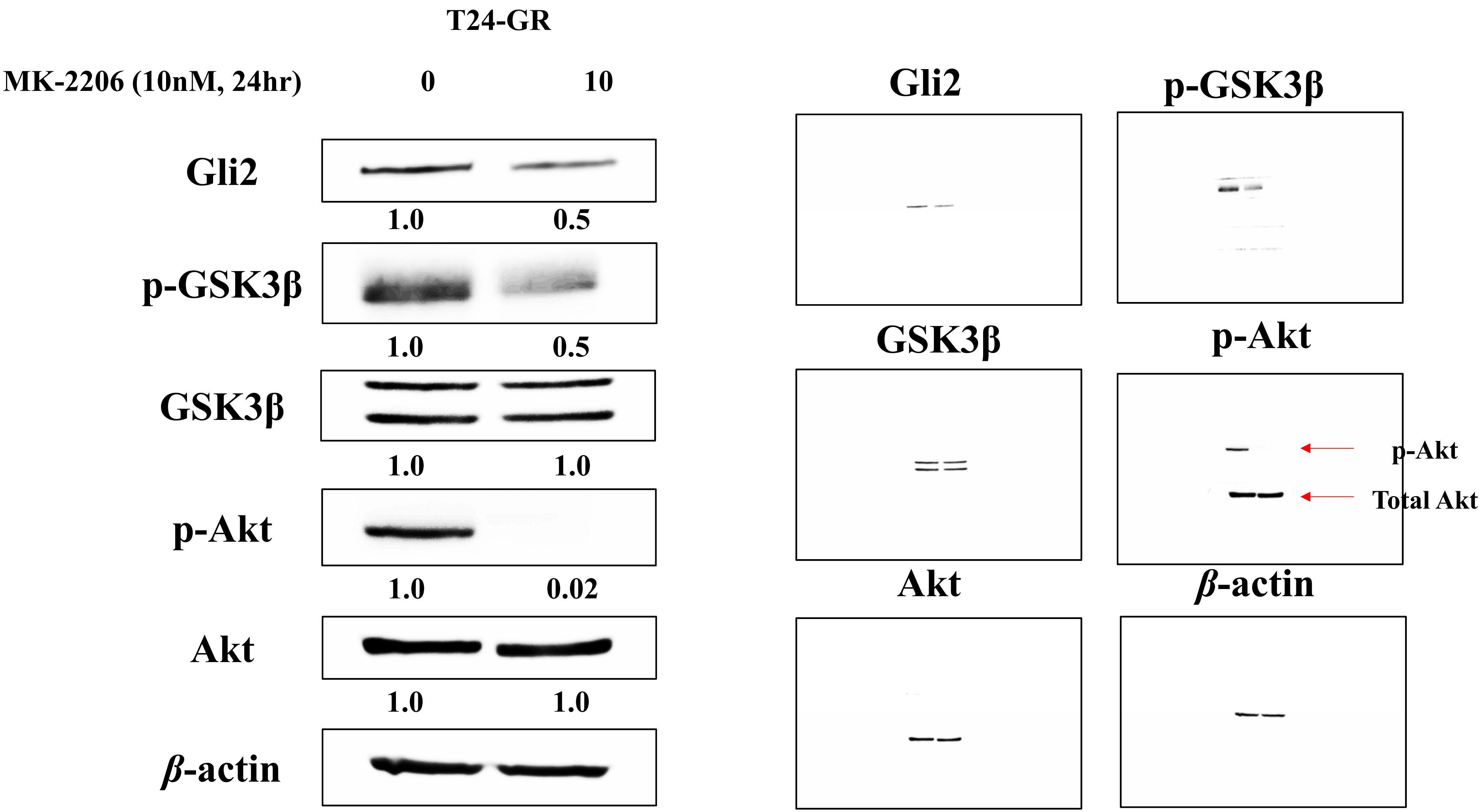
T24-GR
MK-2206 (10nM, 24hr)
0
10
Gli2
1.0
0.5
p-GSK3β
1.0
0.5
GSK3β
1.0
1.0
p-Akt
1.0
0.02
Akt
1.0
1.0
β-actin
Gli2
p-GSK3β
GSK3β
p-Akt
p-Akt
Total Akt
Akt
β-actin
